# TAK1 inhibition mitigates intracerebral hemorrhage-induced brain injury through reduction of oxidative stress and neuronal pyroptosis via the NRF2 signaling pathway

**DOI:** 10.3389/fimmu.2024.1386780

**Published:** 2024-05-02

**Authors:** Jing Zhao, Chunli Chen, Lite Ge, Zheng Jiang, Zhiping Hu, Lihong Yin

**Affiliations:** ^1^ Department of Neurology, Second Xiangya Hospital, Central South University, Changsha, China; ^2^ Clinical Medical Research Center for Stroke Prevention and Treatment of Hunan Province, Department of Neurology, Second Xiangya Hospital, Central South University, Changsha, China

**Keywords:** intracerebral hemorrhage, pyroptosis, reactive oxygen species, oxidative stress, neuroinflammation, NLRP3 inflammasome, therapeutic effects, brain injury

## Abstract

**Introduction:**

Intracerebral hemorrhage (ICH) often triggers oxidative stress through reactive oxygen species (ROS). Transforming growth factor-β-activated kinase 1 (TAK1) plays a pivotal role in regulating oxidative stress and inflammation across various diseases. 5Z-7-Oxozeaenol (OZ), a specific inhibitor of TAK1, has exhibited therapeutic effects in various conditions. However, the impact of OZ following ICH and its underlying molecular mechanisms remain elusive. This study aimed to explore the possible role of OZ in ICH and its underlying mechanisms by inhibiting oxidative stress-mediated pyroptosis.

**Methods:**

Adult male Sprague-Dawley rats were subjected to an ICH model, followed by treatment with OZ. Neurobehavioral function, blood-brain barrier integrity, neuronal pyroptosis, and oxidative stress markers were assessed using various techniques including behavioral tests, immunofluorescence staining, western blotting, transmission electron microscopy, and biochemical assays.

**Results:**

Our study revealed that OZ administration significantly inhibited phosphorylated TAK1 expression post-ICH. Furthermore, TAK1 blockade by OZ attenuated blood-brain barrier (BBB) disruption, neuroinflammation, and oxidative damage while enhancing neurobehavioral function. Mechanistically, OZ administration markedly reduced ROS production and oxidative stress by facilitating nuclear factor-erythroid 2-related factor 2 (NRF2) nuclear translocation. This was accompanied by a subsequent suppression of the NOD-like receptor protein 3 (NLRP3) activation-mediated inflammatory cascade and neuronal pyroptosis.

**Discussion:**

Our findings highlight that OZ alleviates brain injury and oxidative stress-mediated pyroptosis via the NRF2 pathway. Inhibition of TAK1 emerges as a promising approach for managing ICH.

## Introduction

Intracerebral hemorrhage (ICH) remains a life-threatening cerebrovascular disease with poor prognosis (1, 2). Despite constituting only 27.9% of newly occurring strokes, ICH is responsible for over 50% of global disability (3). Unfortunately, effective pharmaceutical interventions aimed at enhancing neurofunctional outcomes of ICH are limited (4). ICH elicits both primary and secondary brain injuries. Primary brain injury mainly results from hematoma enlargement and physical damage to the brain parenchyma, while secondary brain injury leads to persistent and severe neurological complications (1, 5, 6). The mechanisms underlying ICH secondary brain injury are intricate, involving oxidative stress, neuronal inflammation, and cytotoxicity. Pyroptosis plays a vital role in pathophysiological mechanisms underlying secondary brain injury (7). These interlinked mechanisms collectively contribute to brain edema and injury (6, 8).

Pyroptosis is a programmed process of inflammatory cell death, differing from other forms of programmed death, such as necrosis and apoptosis, in both morphology and mechanism and exhibiting severe proinflammatory effects (9). Pyroptosis is characterized by the swift rupture of the plasma membrane, resulting in the release of cellular contents and cytokines, as well as the formation of plasma membrane pores. This process eventually leads to water influx, cellular swelling, and rupture (10). Reactive oxygen species (ROS) play a crucial role in the activation of ICH-induced NOD-like receptor protein 3 (NLRP3) inflammasome. By reducing oxidative stress, the inflammatory response of NLRP3 can be inhibited, thereby indirectly protecting nerve cells from death and further mitigating ICH damage (11, 12). However, the molecular mechanisms underlying the relationship between neuronal pyroptosis and oxidative damage following ICH remain unclear. Therefore, there is an urgent need to explore effective strategies to inhibit ROS accumulation, oxidative stress, and pyroptosis to protect neuronal cells from death after ICH.

Transforming growth factor-β-activated kinase 1 (TAK1) is a member of the mitogen-activated protein kinase kinase kinase (MAP3K) family (13), regulating various cell death types and encompassing a wide array of biological functions (14, 15). TAK1 mediates the production of ROS, exacerbating oxidative damage in different disease models (16, 17). In the central nervous system (CNS), TAK1 is mainly expressed in neurons, where it modulates a variety of intracellular signaling pathways (18). It has demonstrated that TAK1 blockade restrains phosphorylated NF-κB p65 expression and NLRP3 inflammasome activation post-subarachnoid hemorrhage (SAH) (19). Since these intrinsic mechanisms contribute to the main pathophysiology of secondary brain injury, targeting TAK1 may offer an effective treatment for ICH. However, no studies have reported on the effects of TAK1 in ICH-induced neuronal oxidative stress and pyroptosis.

5Z-7-Oxozeaenol (OZ), a specific inhibitor of TAK1, has recently been evaluated in various disease models (20, 21). OZ inhibits NLRP3-mediated neuronal pyroptosis during EBI following SAH (19). However, whether OZ can protect against brain injury after ICH and its potential molecular mechanisms remain unclear. Hence, this study aims to explore the possible role of OZ in ICH and its underlying mechanisms by inhibiting oxidative stress-mediated pyroptosis.

## Materials and methods

### Animals

Adult male Sprague-Dawley rats (8-week-old), weighing 260–300 g, were procured from the Hunan Slack Jingda Laboratory (Changsha, Hunan, China) and housed in the Center Laboratory of Hunan Provincial People’s Hospital. They were maintained under a standard 12-h light/dark cycle and specific-pathogen-free conditions (temperature 18–26°C, relative humidity 40–70%), with ad libitum access to water and food. The Animal Care and Ethics Review Committee of Central South University approved all procedures (IACUC approval No: 2020079). All the animals were treated humanely.

### Rat ICH model establishment

Rats were intraperitoneally administered sodium pentobarbital (40 mg/kg). Type IV collagenase (0.2 U in 2 µL saline; Sigma-Aldrich, USA) was gradually (0.4ul/min) injected into the right basal ganglia over 5 minutes, utilizing a stereotactically inserted microsyringe (10 µL) through the cranial borehole. The coordinates were as follows: 0.2 mm forward from the anterior fontanelle, 3 mm to the right, and 6.0 mm in depth. The needle was left in place for 5 minutes after the injection to prevent backflow, followed by a slow withdrawal of the needle. The pinhole in the skull was sealed using bone wax and the wound was closed. The same procedure was performed in the sham group with an equivalent volume of physiological saline instead of collagenase type IV.

### Drug administration and intracerebroventricular injection

Briefly, the OZ (1 or 3μg; Sigma-Aldrich, USA, # O9890-1MG) dissolved in 2 μl DMSO or equivalent volume of DMSO was administered into the right lateral ventricle at 0.5 h post-ICH ictus, using the following stereotaxic coordinates: 0.8 mm posterior, 1.5 mm right lateral to the bregma, and 4.5 mm ventral to the skull (22, 23). OZ was injected at a flow rate of 0.4 μl/min using a pump.

### Modified neurologic severity scores test

Neurological deficits were evaluated using a modified neurological severity scoring (mNSS) (24) which include motor, sensory and beam balance tests, absent reflexes and abnormal movements (**Supplementary Table 1**). Of the total 18 scores, a higher mNSS score indicates more severe brain injury.

### Immunofluorescence staining

After behavioral testing, the rats were anesthetized with an intraperitoneal injection of pentobarbital sodium. Following cardiac perfusion with 0.9% sodium chloride and 4% paraformaldehyde, the brains were extracted, fixed in 4% paraformaldehyde for 24 h and dehydrated using an alcohol gradient. Coronal paraffin sections with a thickness of 3 μm were obtained from paraffin blocks using a microtome. Subsequent to high-temperature repair and blocking, the sections underwent an overnight incubation at 4°C with the primary antibody, including anti-phospho-TAK1(Thr184/187) (rabbit. # PA5-99340; Thermo Fisher Scientific), anti-Neun (rabbit. # 26978-1-AP; Proteintech), anti-IBA1(mouse. # ab283319; Abcam), anti-glial fibrillary acidic protein (GFAP; mouse. # 60190-1-ig; Proteintech), and anti-GSDMD (rabbit. # 20770-1-AP; Proteintech). After three washes in phosphate buffer solution (PBS), the sections were exposed to appropriate conjugated secondary antibodies: CoraLite488-conjugated Affinipure Goat Anti-Mouse IgG([H+L] SA00013-1; Proteintech) and CoraLite594-conjugated Goat Anti-Rabbit IgG([H+L] #SA00013-4; Proteintech), followed by counterstaining with DAPI for 10 min in the dark. Images were captured using a fluorescence microscope.

### Western blotting

The brain tissue around the hematoma, including the sensory motor cortex of the hemorrhaged brain after ICH was lysed using RIPA buffer containing proteinase inhibitors. Nuclear proteins were extracted using the Nucl-Cyto-Mem Preparation Kit (#P1201, Applygen, China). The BCA Protein Assay Kit was used to evaluate the protein concentration. Proteins were separated by sodium dodecyl sulfate-polyacrylamide gel electrophoresis (SDS-PAGE), transferred to polyvinylidene fluoride (PVDF) membranes, and blocked with 5% BSA for 1.5 h. Subsequently, western blotting (WB) was conducted with an overnight incubation at 4°C using the corresponding primary antibodies, including P-TAK1 (ab109404, Abcam, USA), TAK1 (ab109301, Abcam, USA), ZO-1 (21773-1-AP, Proteintech, USA), Occludin (27260-1-AP, Proteintech, USA), GSDMD (20770-1-AP, Proteintech, USA), NLRP3 (19771-1AP, Proteintech, USA), ASC (ab180799, Abcam, UK), caspase1 (ab179515, Abcam, UK), IL-1β (16806-1-AP, Proteintech, USA), IL-18 (10663-1-AP, Proteintech, USA), NRF2 (80593-1-RR, Proteintech, USA), HO-1 (ab189491, Abcam, UK), NQO1 (ab80588, Abcam, UK), β-actin (66009-1-Ig, Proteintech, USA), LaminB1 (66095-1-Ig, Proteintech, USA), P62 (AF5384, Affinity Biosciences, USA) and KEAP1 (60027-1-Ig, Proteintech, USA). The target protein signals were normalized to β-actin intensity for standardization. Lamin B1 served as a loading control for nuclear proteins. The membranes were then washed three times with TBST (10 min each) and further incubated for 1.5 h with the secondary antibody at room temperature. Finally, the relative protein expression intensity was analyzed using the ImageJ software.

### Nissl staining

Nissl staining was performed to assess the neuronal damage and loss. After paraffin embedding and sectioning, the brain tissues were stained with a 1% toluidine blue reagent. The number of apoptotic neurons was assessed using ImageJ software.

### Measurement of ROS level

DHE (D7008, Sigma-Aldrich, Shanghai), an oxidative fluorescent stain, was employed to assess ROS levels in rat brain tissue. Frozen brain tissue sections, 6-8 µm thick, were incubated with 50 µl of DHE (1:1000, 30 min) at 37°C for 30 min, shielded from light. Following this, the sections were treated with DAPI for 10 minutes. Subsequently, the sections were rinsed thrice with PBS. DHE staining images were captured using a light microscope, and the DHE-positive cells were quantified using ImageJ software.

### Transmission electron microscopy

Cerebral cortex tissues were fixed in 2.5% glutaraldehyde for 8 h and rinsed with PBS. Secondary fixation was performed using 1% osmic acid solution for at least 1 h. The tissues underwent a graded dehydration series using ethanol (30%, 50%, and 70% uranyl acetate and 80%, 95%, and 100% uranyl acetate). Following dehydration, the samples were embedded in epoxy resin, cut into ultrathin sections, and stained with uranyl acetate and lead citrate. Images were captured using a transmission electron microscope (TEM) (#JEM1400, Japan).

### Measurement of lipid peroxidation and superoxide dismutase

The supernatants from the brain tissue homogenates were harvested. The level of LPO (Lipid Peroxidation) was detected using the lipid peroxidation assay kit (A106-1, Nanjing Jiancheng Bioengineering Institute, Nanjing, China). For measuring the total superoxide dismutase (SOD) concentration in brain tissues, a SOD assay kit was used employed (A001-3, Nanjing Jiancheng Bioengineering Institute, Nanjing, China). LPO and SOD levels were calculated based on the protein concentration and OD value, following the manufacturer’s instructions.

### Statistical analysis

All quantitative data were analyzed using GraphPad Prism 8.0 and presented as means ± standard error of the mean (SEM). Differences between the two groups were assessed using Student’s t-test. One-way analysis of variance (ANOVA) followed by Tukey’s post-hoc test was used to compare three or more groups. P < 0.05 was considered statistically significant.

## Results

### Time course and spatial expressions of TAK1 in the ipsilateral cortex after ICH

**Figure 1:**
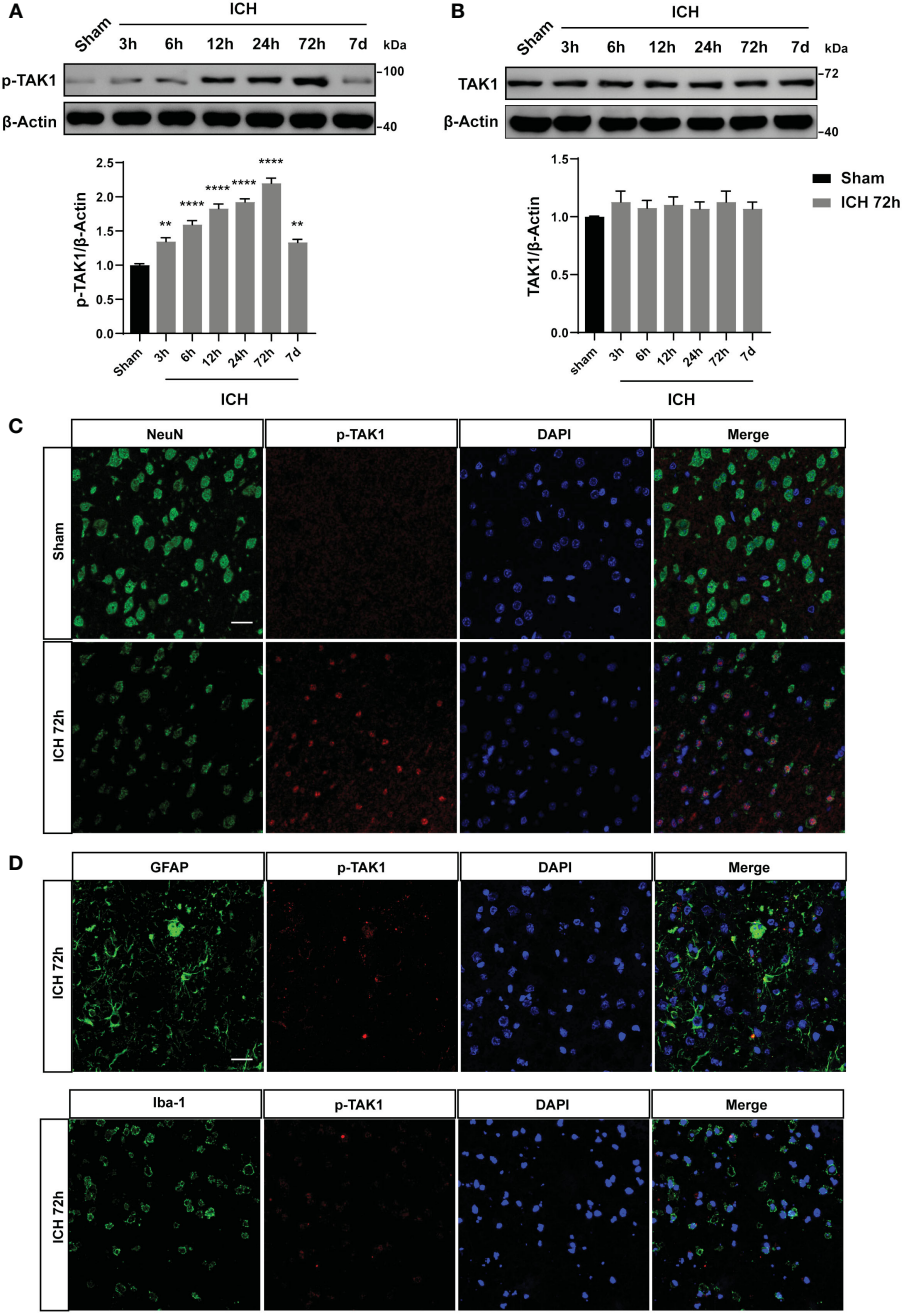
Expression pattern and cellular localization of TAK1 in the ipsilateral cortex post-ICH. A: Representative western blot bands of time course and quantitative analysis of p-TAK1 at sham, 3 h, 6 h, 12 h, 24 h, 72 h, and 7d post-ICH. B: Western blot analysis of TAK1 expression in rat brain post-ICH. C, D: Co-localization of p-TAK1 with cell-type markers. P-TAK1(red) co-localized with NeuN-positive neurons (green) both in Sham and ICH 72 h groups (n = 3). Nuclei were stained with DAPI (blue). Data are expressed as mean ± SEM; ** P <0.01, ****P < 0.0001 vs Sham group. Scale bar = 25 μm

We detected the temporal profiles of p-TAK1 and TAK1 protein expression using WB analysis in the ipsilateral brain tissue around hematoma include sensory motor cortex of hemorrhaged brain after ICH. The results indicated a significant increase in p-TAK1 expression at 12 h, reaching its peak at 72 h, and subsequently decreasing at 7d after ICH compared to the sham group (**Figure 1A**). Conversely, as depicted in **Figure 1B**, the total expression of TAK1 did not differ among the groups. Having elucidated the temporal orientation of TAK1, we explored its spatial orientation. Double immunofluorescence staining of p-TAK1 with NeuN, Iba1, and GFAP was conducted to determine its cellular localization 72h post-ICH. The results demonstrated that p-TAK1 signals were primarily observed in neurons, rather than in GFAP-positive astrocytes or Iba1-positive microglia. Additionally, the number of p-TAK1 positive neurons increased in ICH rats (72h) compared to sham rats (**Figures 1C, D**).

### TAK1 inhibition improves neurobehavioral function, BBB disruption

**Figure 2:**
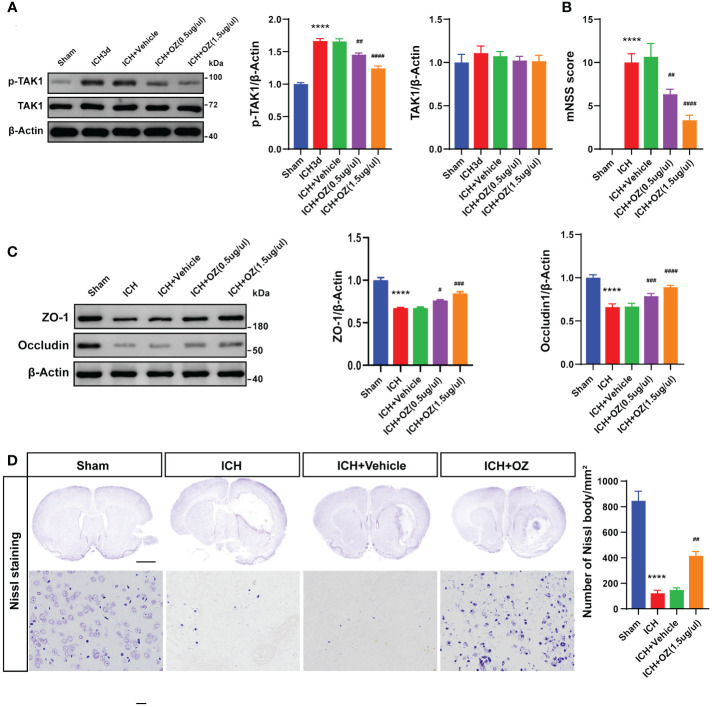
TAK1 inhibition improves neurological deficits and attenuates BBB disruption post-ICH. A: Western blots analysis of p-TAK1 and TAK1 in ipsilateral cortex from Sham, ICH, ICH + Vehicle, and ICH+OZ (0.5 μg/μL and 1.5 μg/μL) groups and quantification analysis of the proteins. B: Neurological scores assessment of rats in each group (n = 6). C: Western blot images and densitometric analysis of tight junction proteins, including ZO-1 and Occludin. D: Nissl staining of rat brains in the four groups (sham, ICH, ICH +vehicle, ICH +OZ). Scale bar = 1 mm; bar = 25 μm and number of Nissl bodies (n = 3). Data are expressed as mean ± SEM; ****P < 0.0001 vs Sham group; #P < 0.05, ##P < 0.01, ###P < 0.001 and ####P <0.0001vs ICH +vehicle group.

To determine the biological function of TAK1 post-ICH, OZ, a specific inhibitor of TAK1, was administered to attenuate the activation of TAK1 (**Figure 2A**). Two doses of OZ were tested to determine the optimal dose for attenuating ICH-induced brain injury. Neurological function was assessed using the mNSS after TAK1 suppression in rats. At 72 h, significant neurological deficits were observed in the ICH group compared to the sham group. Administration of 0.5 μg/μL of OZ did not notably improve neurological performance, whereas OZ (1.5 μg/μL) significantly improved neurological outcomes caused in ICH rats compared to the ICH + vehicle group (**Figure 2B**).

To further validate the treatment efficacy of OZ and evaluate BBB permeability, the tight junction proteins (ZO-1 and Occludin) were assessed 72 h after ICH. Reduced expression of tight junction proteins was observed in the ipsilateral cortex of ICH rats compared to sham-operated rats. Application of OZ (1.5 μg/μL) significantly alleviated BBB permeability damage post-ICH (**Figure 2C**). Therefore, OZ at the dosage of 1.5 μg/μL was selected for subsequent studies. While known for its ability to effectively inhibit TAK1, OZ also effectively inhibits a variety of other kinases, including VEGFR2, PDGFR-β, and MEK1 (20, 25–27). Therefore, we have conducted a series of Western Blot experiments to assess the effects of OZ treatment on these pathways in the context of ICH. Our research findings indicate that other cell signaling pathways in ICH, such as VEGFR2, PDGFR-β, and MEK1 signaling, are not affected by OZ treatment (**Supplementary Figure S1**). Considering the increased TAK1 expression in neurons after ICH, Nissl staining was performed. The results showed a reduction in the number of Nissl bodies and severe neuronal damage in the ICH and ICH +vehicle groups, while TAK1 inhibition upregulated the number of Nissl bodies (**Figure 2D**).

### Downregulation of TAK1 attenuates neuronal pyroptosis post-ICH

**Figure 3:**
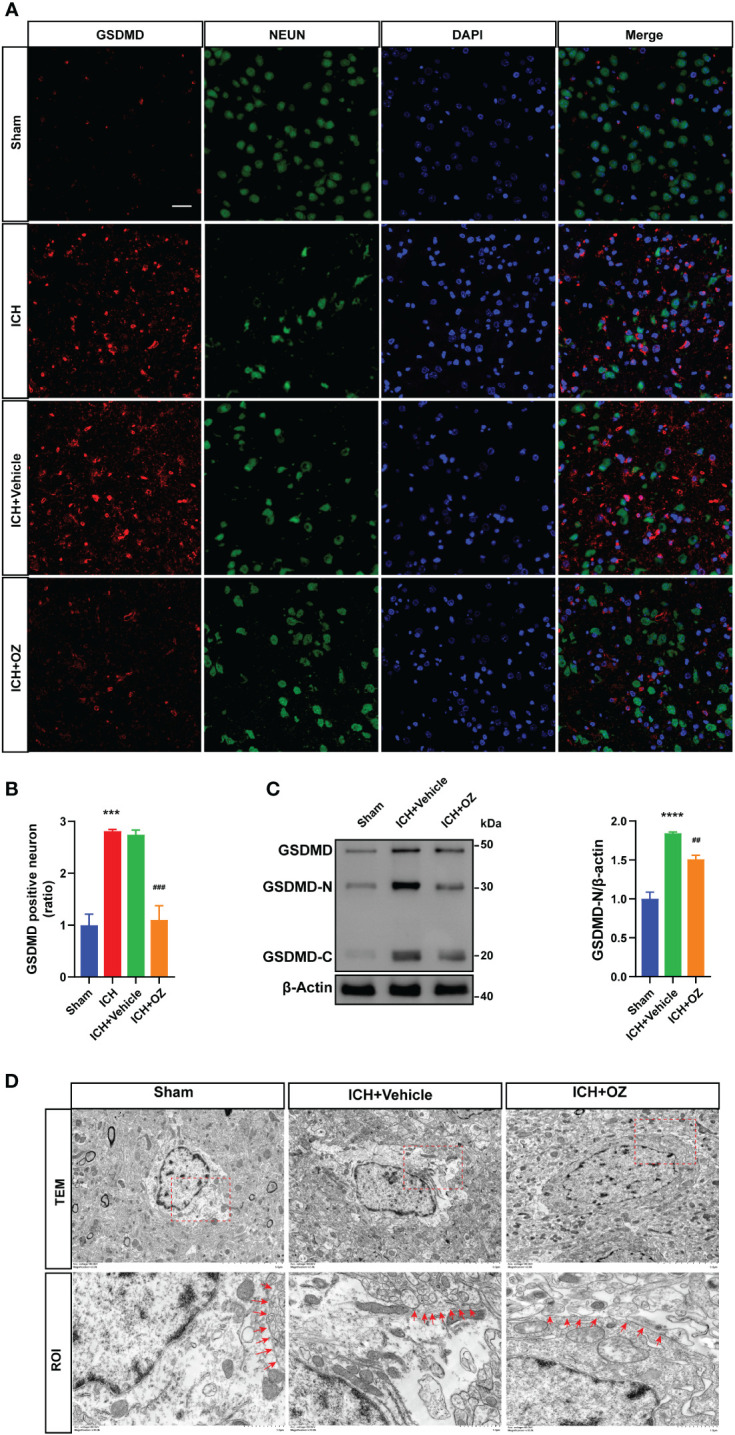
OZ treatment reduces neuronal pyroptosis post-ICH. A: Co-localization of GSDMD (red) with neurons (NeuN, green) in the perihematomal area post-ICH. Scale bar: 25μm. B: Quantification of GSDMD-positive neurons. C: Immunoblot analysis of GSDMD expression in treated rats. D: Transmission electron microscopy (TEM) of cortical neurons in rat brain tissue. Images of selected regions (red squares) are shown at a higher magnification. Red arrows indicate the neuronal membrane pores of different treatment groups. The membrane of neurons in the ICH+ Vehicle groups is broken, while that in the ICH + OZ group is intact (n = 3). Scale bar: 5 μm; Scale bar: 1μm. The values are the mean ± SEM. ***P < 0.001, ****P < 0.0001 vs Sham group; ##P < 0.01, ###P < 0.001 vs ICH +vehicle group.

To further investigate the therapeutic mechanism of TAK1 in ICH-induced cerebral injury, we detected the expression of the N-terminal domain of gasdermin D (GSDMD) in ipsilateral cortex tissues by immunofluorescence staining (**Figure 3A**). The results revealed a significant increase in GSDMD fluorescence intensity due to ICH, which was subsequently reversed by OZ treatment (**Figure 3B**). Additionally, we evaluated the expression of pyroptosis-related proteins, including GSDMD-N terminal, the activated form of GSDMD, using western blotting. The findings indicated a marked increase in GSDMD activity during ICH compared to the sham group. Notably, OZ reversed the ICH-induced alterations in GSDMD-N, aligning with the immunofluorescence results (**Figure 3C**). Pyroptosis is characterized by the formation of plasma membrane pores. Therefore, TEM was employed to observe changes in the pores formed by GSDMD-N on neuronal membranes in different treatment groups. As depicted in **Figure 3D**, more GSDMD membrane pores were observed in neurons in the ICH group than in the sham group, while fewer membrane pores were observed in the OZ-treated group than in the vehicle-treated group.

### Inhibition of TAK1 prevents neuronal pyroptosis by the NLRP3 inflammasome

**Figure 4:**
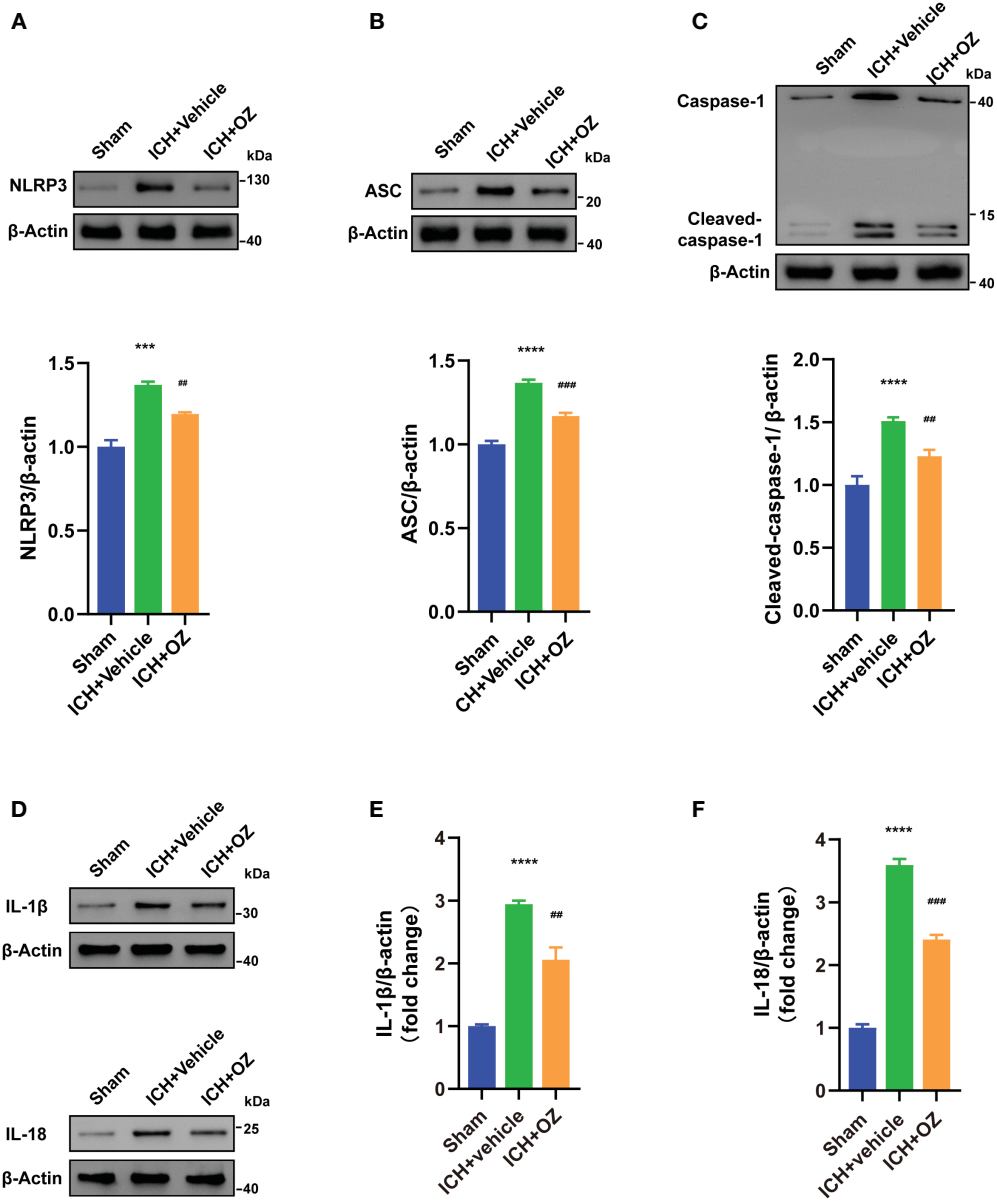
OZ inhibits NLRP3 inflammasome-mediated pyroptosis. **(A)** Immunoblotting signals and quantification of NLRP3 from Sham, ICH + vehicle, and ICH + OZ groups. **(B)** Western blot and quantitative analysis of ASC in the ipsilateral cortex post-ICH. **(C)** Western blot analysis of cleaved caspase-1 protein expression in rat brain tissue from each group. **(D–F)** Representative western blot bands and quantitative analyses of IL-1β, IL-18 (n= 3) The values are the mean ± SEM. ***P < 0.001, ****P < 0.0001 vs Sham group; ##P < 0.01, ###P < 0.001 vs ICH +vehicle group.

Next, we investigated whether NLRP3 inflammasome activation was involved in TAK1-induced neuronal pyroptosis. We used western blotting to visualize signaling proteins, including NLRP3 (**Figure 4A**), ASC (**Figure 4B**), and cleaved caspase-1 (**Figure 4C**). Furthermore, pyroptosis proteins, including IL-1β and IL-18 (**Figures 4D–F**), were also detected using western blot. The results indicated that ICH-injured rats demonstrated a marked increase in NLRP3, ASC, cleaved caspase-1, IL-1β and IL-18 in the ipsilateral cortex compared to sham rats, whereas OZ treatment effectively decreased the expression of these proteins.

### OZ inhibits oxidative stress and activates the NRF2 signaling

**Figure 5:**
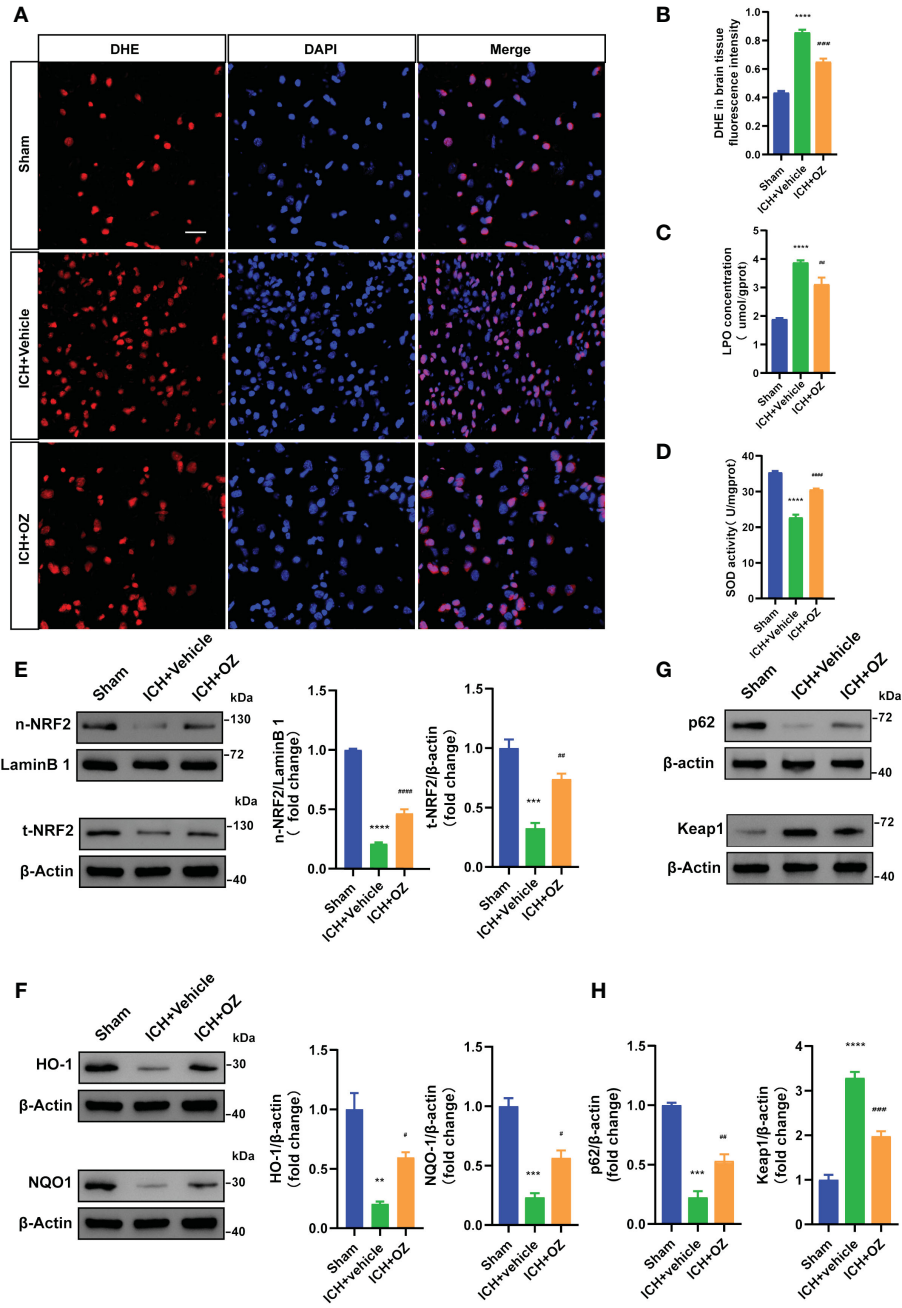
OZ exhibits antioxidative effects by activating NRF2 signaling pathways. **(A)** Representative images for DHE staining of rats’ brain tissue sections (n = 3). **(B)** Quantitative analysis of DHE fluorescence intensity. **(C)** Measurement of LPO content in brain tissue (n = 4). **(D)** Assessment of SOD activity in brain tissue (n = 4). **(E–H)** Representative immunoblot images and quantitative analysis of NRF2, HO-1, NQO1, P62 and KEAP1 in rats brain tissue (n = 3). The values are the mean ± SEM. **P< 0.01, ***P < 0.001 and ****P < 0.0001 vs Sham group; #P < 0.05, ##P < 0.01, ###P < 0.001 and ####P <0.0001vs ICH +vehicle group.

To determine whether OZ treatment decreased oxidative stress in ICH-induced cerebral injury, we examined ROS deposition levels in frozen sections using DHE staining, which directly represents ROS generation. As shown in **Figures 5A, B**, ICH significantly increased ROS levels, which were decreased following OZ treatment. LPO and SOD levels were evaluated to assess oxidative stress. The LPO content was significantly higher in the ICH+Vehicle group than in the sham group. Interestingly, LPO levels significantly decreased in the ICH+OZ group (**Figure 5C**). Additionally, the activity of the major antioxidant enzyme SOD, a standard marker of antioxidative stress, was elevated in the brain tissue of the ICH + OZ group compared to that in the ICH + vehicle group (**Figure 5D**). These results suggest that antioxidant levels in rats in the OZ treatment group recovered to a certain extent. NRF2 is important for maintaining cellular redox homeostasis. Therefore, to further investigate the mechanisms underlying the ability of OZ to inhibit oxidative stress, we used western blotting to detect the expression of oxidative stress-related proteins, including NRF2 nuclear translocation and the downstream antioxidant genes HO-1, NQO1. The nuclear NRF2 and total NRF2 decreased in the model group rats (**Figure 5E**, P<0.001); however, OZ treatment reversed this trend. Furthermore, as expected, the levels of HO-1 and NQO1 were substantially reduced in ICH rats compared to sham rats and reversed in the OZ treatment groups (**Figure 5F**, p < 0.01). Research has found that P62 activates NRF2 via direct interaction with Kelth-like ECH-associated protein 1 (KEAP1), leading to dissociation of NRF2 from KEAP1, which in turn initiates the expression of downstream antioxidant genes in the nucleus. It has been reported that TAK1 could regulate P62 and release NRF2 from KEAP1, resulting in the increased translocation of NRF2. However, it remains unknown whether the effect of OZ on NRF2 activation after ICH is associated with P62. Thus, Western blotting was conducted to detect the expression of P62 and KEAP1 after ICH. As shown in **Figures 5G, H**, the expression level of P62 was significantly decreased in the ICH + Vehicle group than in the control group, while OZ treatment significantly upregulated P62 expression. Furthermore, protein levels of KEAP1 were significantly upregulated after ICH, which was abolished by treatment with OZ. Based on these data, we speculated that NRF2-related signaling pathways might be related to the protective effect of OZ in ICH. Pharmacological inhibition of TAK1 with OZ may activate NRF2 signaling pathway by increasing the expression level of P62 protein.

## Discussion

**Figure 6:**
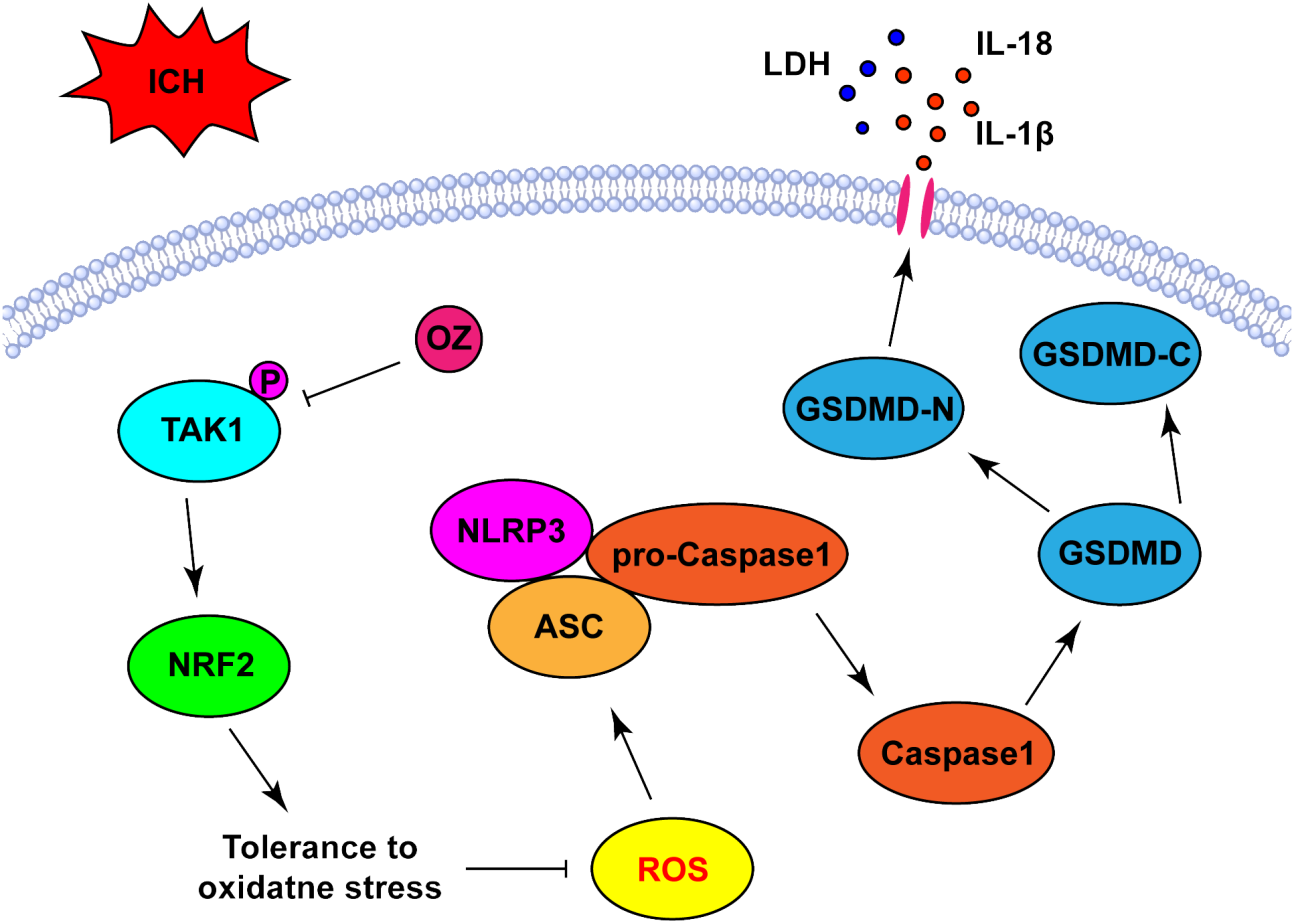
Schematic diagram the possible mechanisms of OZ action post-ICH. ICH significantly upregulated the expression level of p-TAK1 in neurons, indicating the activation of TAK1 in neurons post-ICH. Pharmacological blockade of TAK1 with OZ attenuated neurological impairments and BBB disruption. OZ remarkably reduced neuronal pyroptosis by inhibiting NLRP3 inflammasome activation. The mechanism of OZ in inhibiting NLRP3 inflammasome activation may be related to promoting NRF2 nuclear translocation, thereby attenuating ROS production and oxidative stress.

In this study, we verified that OZ reduced ROS production and oxidative stress by promoting NRF2 nuclear translocation, which was accompanied by the further suppression of the NLRP3 activation-mediated inflammatory cascade and pyroptosis. These results suggest that OZ increases antioxidant capacity to mitigate ICH-induced oxidative stress by regulating the NRF2 signaling pathway (**Figure 6**).

Upon the occurrence of ICH, the activation of the hemoglobin-heme-iron metabolic axis is initiated, leading to increased generation of ROS (28). ROS has been demonstrated to induce NLRP3 inflammasome activation (29, 30). Pyroptosis necessitates inflammasome activation. Currently, the most widely studied inflammasome is NLRP3, which mediates the emergence of pyroptosis in various diseases, including dilated cardiomyopathy, ischemic stroke, myocardial ischemia, cerebral hemorrhage, inflammation, and tumors diseases (31–34). NLRP3 inflammatosomal activation converts precursor caspase-1 to cleaved caspase-1, which further cleaves the precursor of IL-1β and IL-18 into bioactive mature pro-inflammatory cytokines. Additionally, it cleaves GSDMD to form cellular membrane pores, subsequently releasing pro-inflammatory intracellular contents that amplify the inflammatory response (35, 36). Studies have demonstrated that the administration of ROS scavenger decreases NLRP3 and its downstream pyroptosis protein levels in a ROS-dependent manner, thereby alleviating neuroinflammation and secondary brain injury post-ICH (28, 37, 38). In light of this, the specific mechanism by which TAK1 indirectly activates the NLRP3 inflammasome after ICH involves increasing intracellular ROS formation.

Pharmacological strategies targeting the NLRP3 inflammasome have been developed; however, recognizing inflammasomes as a fundamental mechanism of innate immunity suggests that an appropriate defense system may be more essential than a specific NLRP3 inhibitor (39). The NRF2 signaling pathway serves as an intrinsic cytoprotective mechanism against oxidative stress by regulating the expression of numerous antioxidant factors to eliminate excess ROS (40). It also has been shown to counteract NLRP3 inflammasome-mediated pyroptosis by reducing ROS-mediated oxidative stress (41, 42). To date, preclinical and clinical studies on NRF2 activators against NLRP3 inflammasome activation have made some progress in CNS disorders (39). NRF2 is a crucial transcription factor responsible for maintaining the redox balance. Under normal physiological conditions, NRF2 binds to KEAP1, and its function is inhibited in the cytoplasm. Oxidative stress reduces KEAP1 expression, thereby promoting the free NRF2 translocate into the nucleus (43). There, it binds to the antioxidant response element and subsequently modulates the activity of antioxidant enzymes (44, 45). Notably, NQO1, a downstream effector of NRF2 signaling, plays a critical role in ROS scavenging and regulation of ROS generation (46). In addition to the classical activation pathway of KEAP1-NRF2, the untypical mechanism of NRF2 activation, i.e., the autophagy lysosome pathway triggered by autophagy dysfunction, is also involved in mediating oxidative stress. The transcription of autophagic receptor P62 can be activated by oxidative stress and inhibition of autophagy, resulting in large accumulation of P62 in the cytoplasm. The aggregated P62 directly interacts with the NRF2 inhibitory protein KEAP1 to maintain activation of NRF2, leading to upregulation of gene transcription encoding antioxidant enzymes, thereby protecting cells from oxidative damage. Research has shown that P62 competes with NRF2 to bind to KEAP1, allowing P62 to chelate KEAP1 into autophagosome, consequently preventing KEAP1-mediated NRF2 degradation and activating the NRF2 pathway (47, 48). In addition, NRF2 in the nucleus promotes overexpression of the P62 gene, forming a positive feedback axis of P62-KEAP1-NRF2, leading to sustained activation of NRF2 to eliminate ROS (49). A previous study revealed that TAK1 upregulates the binding of KEAP1 with P62/SQSTM1 through mediating S351 phosphorylation of P62/SQSTM1, which facilitates KEAP1 degradation and upregulates NRF2 in the absence of exogenous oxidative stress. This mechanism provides antioxidant protection under steady-state conditions in the intestinal epithelium (50). However, recent research has shown that 4F5C-QAME directly targeting TAK1 activated NRF2/HO-1 signaling pathway by alleviating the interaction with KEAP1-regulated NRF2 activation, thereby reducing oxidative stress (51), the regulatory effect of TAK1 on NRF2 activation through alleviating the interaction with KEAP1 under exogenous oxidative stress was revealed for the first time. Herein, our data indicated that OZ treatment attenuates the activation of TAK1 increased the protein expression of P62, nuclear NRF2, HO-1, NQO1 and suppressed KEAP1. The differences between these studies might be due to TAK1 having different regulatory effects in different disease models, animal species and measurement time points, with harmful or beneficial effects depending on factors such as stimulus and cell type.

In addition to ROS, such as extracellular ATP, K+ efflux, and endosomal rupture, endogenous danger-associated molecules and mitochondrial dysfunction could trigger NLRP3 inflammasome activation (36, 52). Hindi et al. indicated that TAK1 plays a critical role in the regulation of skeletal muscle mass and oxidative metabolism. TAK1 activation can induce the accumulation of dysfunctional mitochondria and oxidative damage in skeletal muscle (53). We suggest that TAK1 also affects mitochondrial dysfunction by triggering NLRP3 inflammation. However, further studies are required to determine whether other molecular targets are involved in this modulation. Evidence has shown that mitochondrial dysfunction can promote excessive ROS generation to increase total cellular oxidative stress (54). As this study mainly focused on TAK1 for total intracellular ROS, further investigation into the involvement of mitochondrial stress in OZ is warranted in the future study.

Our study had several limitations. Firstly, while our research elucidated the mechanisms by which TAK1 regulates NLRP3/caspase-1-dependent pyroptosis, we cannot exclude the potential contribution of noncanonical pyroptotic pathways, such as the NLRP1 inflammasome and activated caspase-4/5/11 in ICH. Secondly, although we have investigated and found that OZ has no effect on other signaling pathways, such as MEK1/2, VEGFR2, and PDGFR-β, in the context of ICH, further work is needed. Specifically, we need to examine the effect of TAK1 inhibition through TAK1-specific knockdown in ICH to confirm the specific selectivity of OZ for TAK1. Thirdly, we solely assessed the neuroprotective effects of OZ on neural pyroptosis and inflammation. Additional research is needed to explore other pathological processes, including brain edema, calcium overload, and ferroptosis. Further experiments are required to answer these questions.

## Conclusion

In conclusion, this study focused on the role of OZ in brain injury and investigated its underlying mechanisms. Our findings demonstrated that OZ alleviates brain injury and oxidative stress-mediated pyroptosis via the NRF2 pathway. Collectively, our research suggests that OZ plays a neuroprotective role in ICH and that TAK1 may serve as a therapeutic target for ICH.

## Data availability statement

The original contributions presented in the study are included in the article/**Supplementary Materials**, further inquiries can be directed to the corresponding author/s.

## Ethics statement

The animal study was approved by The Animal Care and Ethics Review Committee of Central South University (IACUC approval No: 2020079). The study was conducted in accordance with the local legislation and institutional requirements.

## Author contributions

JZ: Conceptualization, Resources, Supervision, Writing – review & editing, Data curation, Methodology, Software, Writing – original draft. CC: Methodology, Software, Writing – review & editing. LG: Methodology, Software, Writing – review & editing. ZJ: Methodology, Software, Writing – review & editing. ZH: Writing – review & editing, Funding acquisition, Project administration. LY: Writing – review & editing, Conceptualization, Data curation, Methodology, Resources, Software, Supervision, Writing – original draft.
