# Supplementary material for: TAK1 inhibition mitigates intracerebral hemorrhage-induced brain injury through reduction of oxidative stress and neuronal pyroptosis via the NRF2 signaling pathway
Source: Front Immunol. 2024 May 2;15:1386780. doi: 10.3389/fimmu.2024.1386780 (PMC11096530; doi:10.3389/fimmu.2024.1386780)
Supplement: Supplementary file 1 [file DataSheet_1.docx]

**Full unedited gel/blot for Figure 1A in the manuscript.**


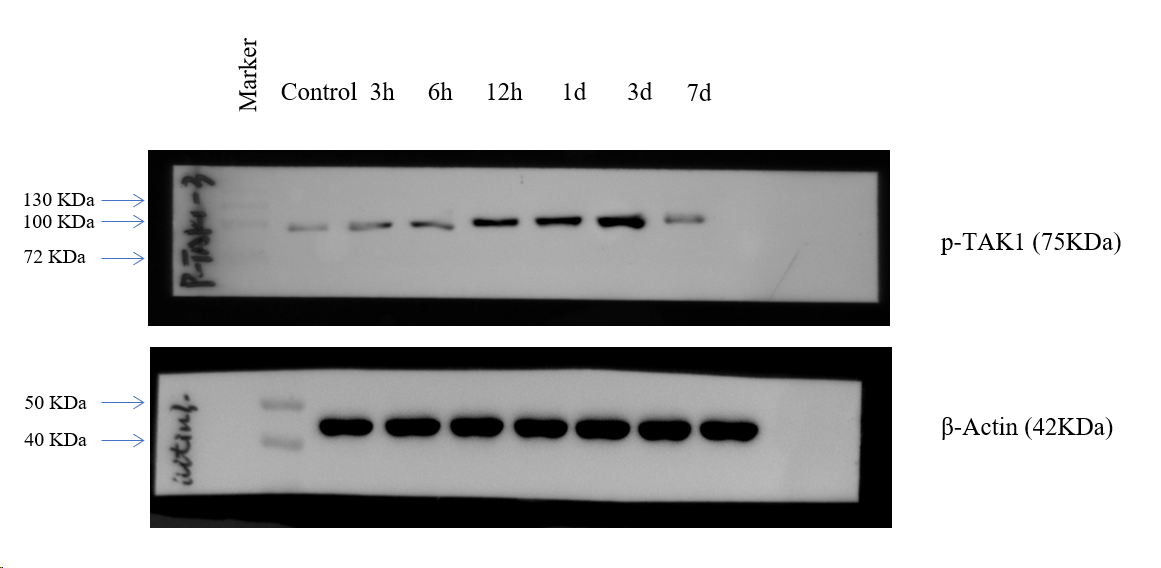


**Full unedited gel/blot for Figure 1B in the manuscript.**

**
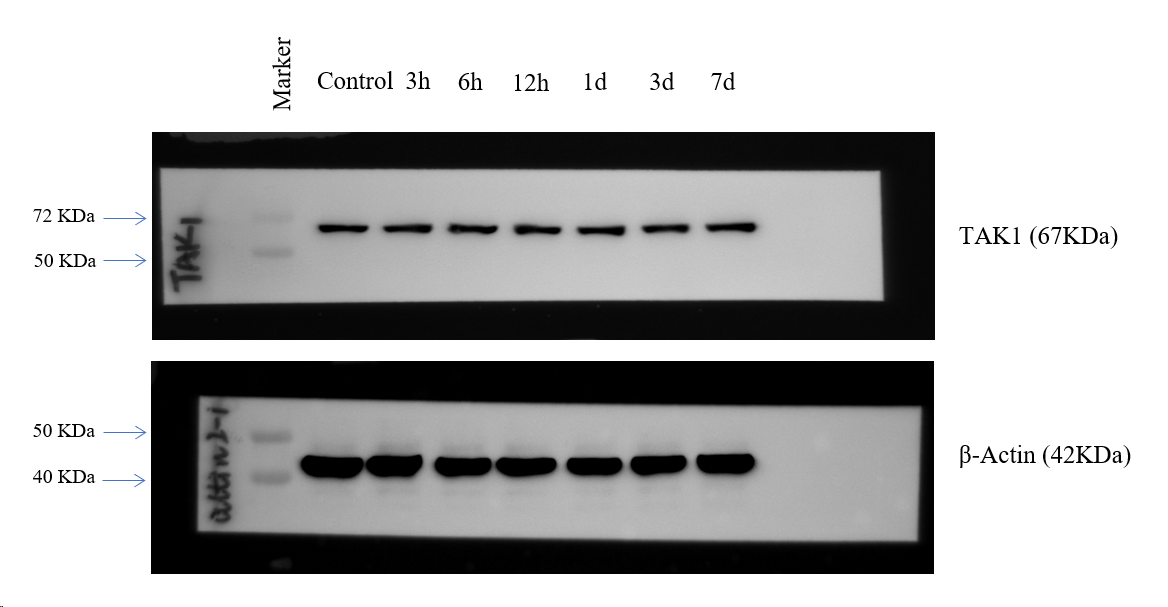
**

**Full unedited gel/blot for Figure 2A in the manuscript.**

**
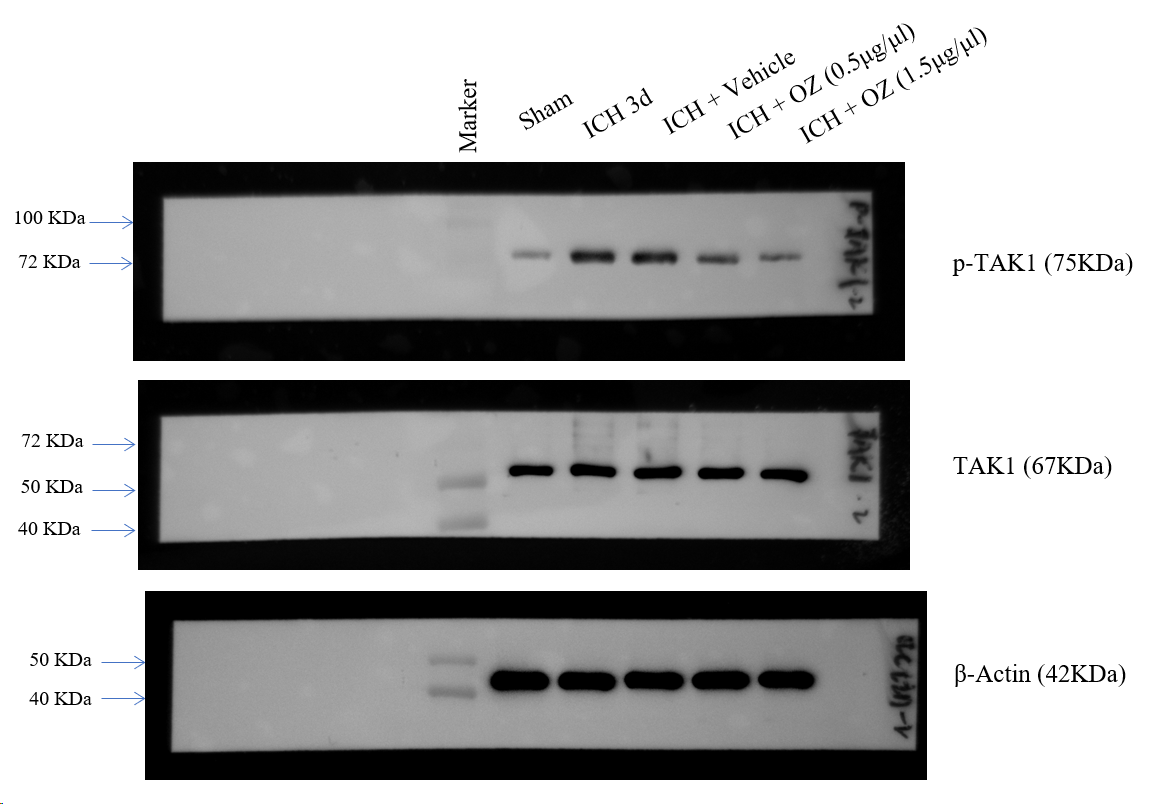
**

**Full unedited gel/blot for Figure 2C in the manuscript.**


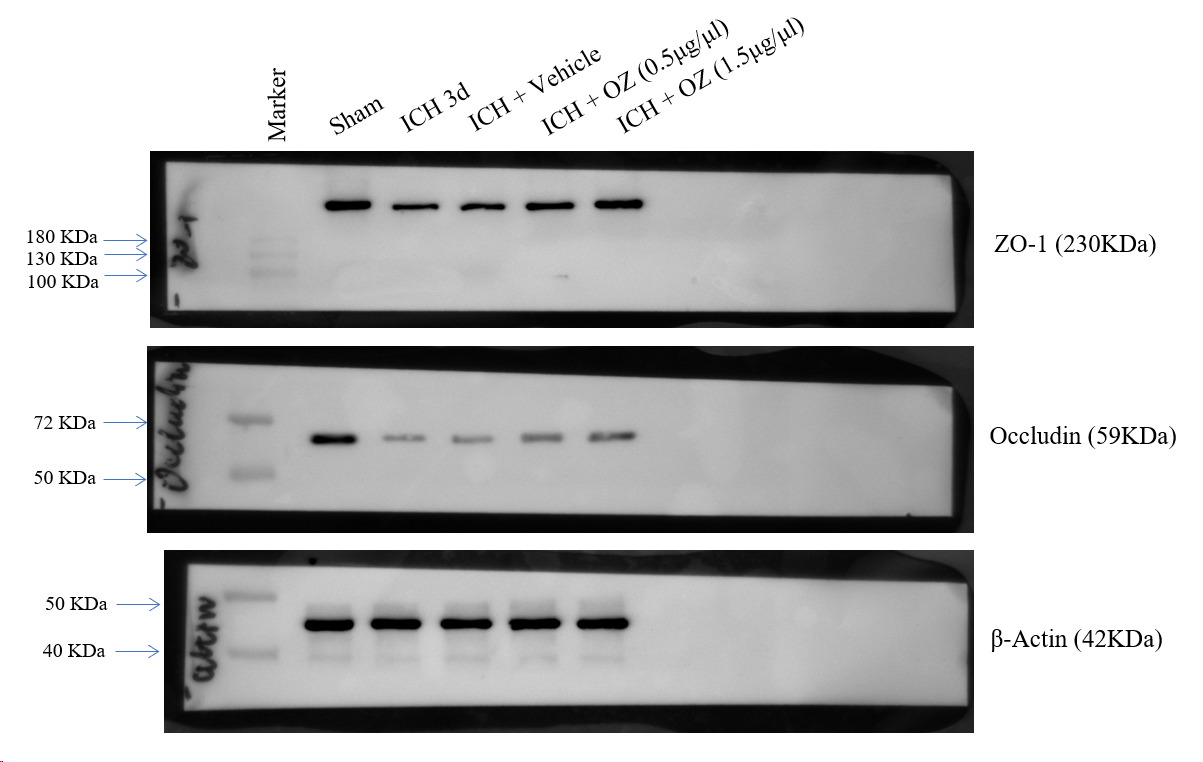


**Full unedited gel/blot for Figure 3C in the manuscript.**

**
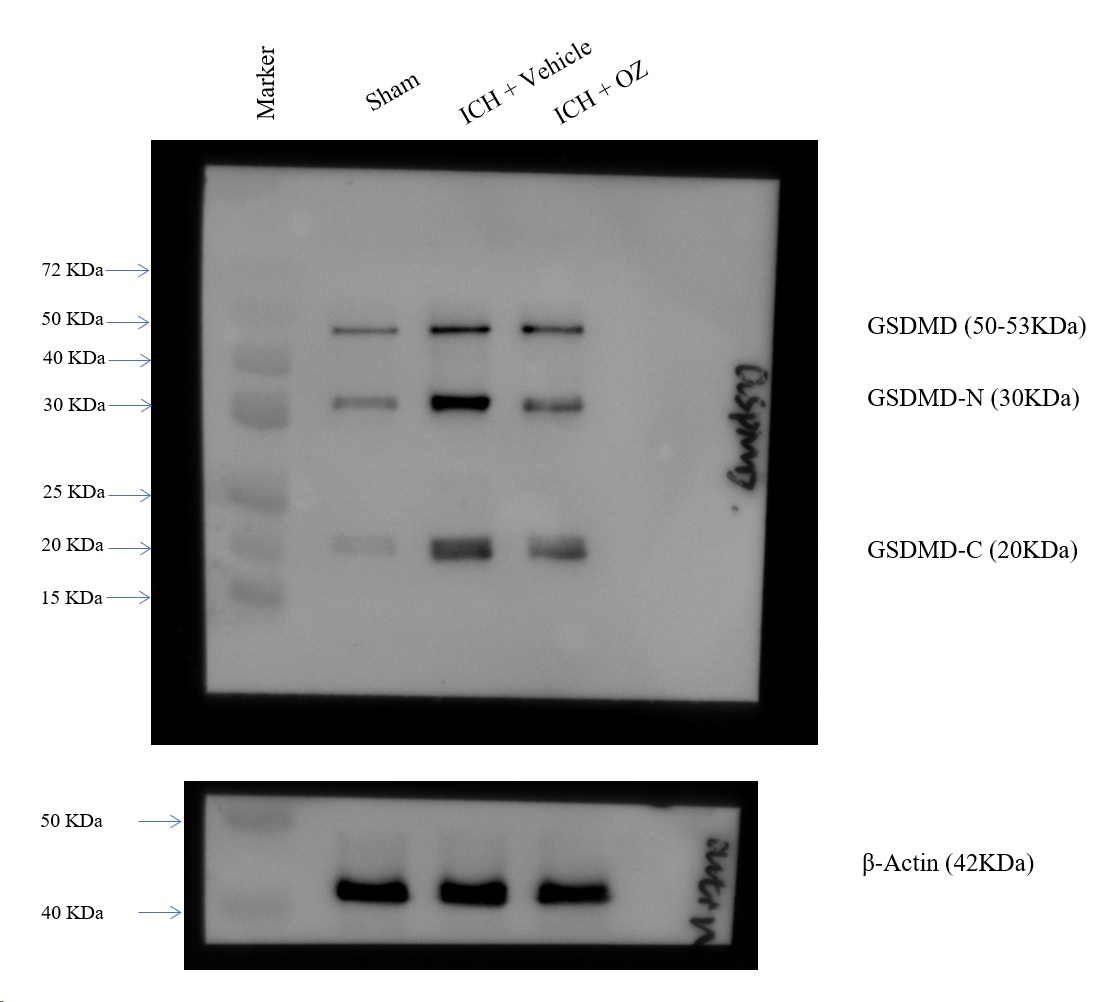
**

**Full unedited gel/blot for Figure 4A in the manuscript.**

**
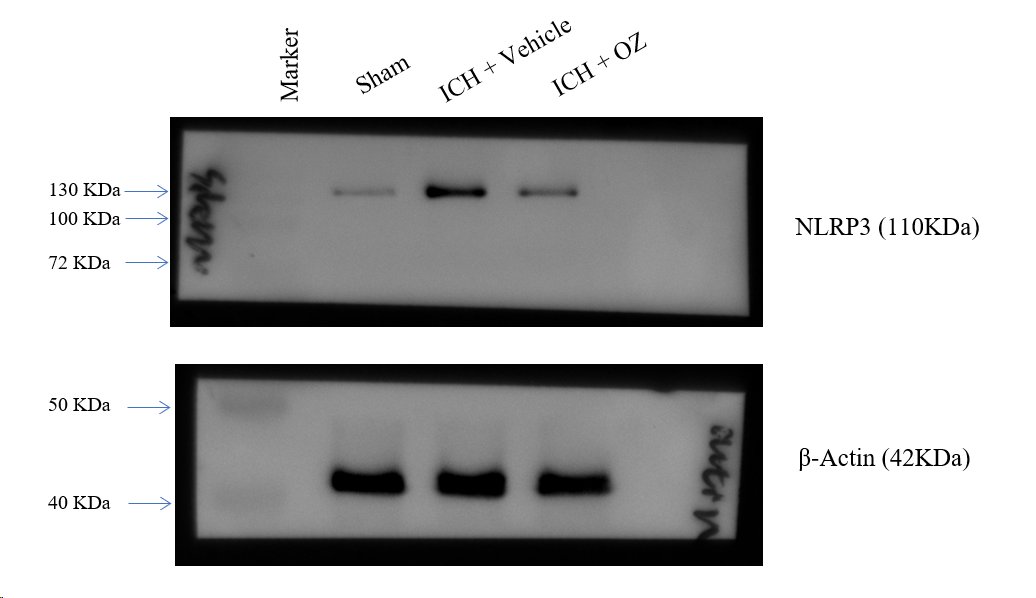
**

**Full unedited gel/blot for Figure 4B in the manuscript.**

**
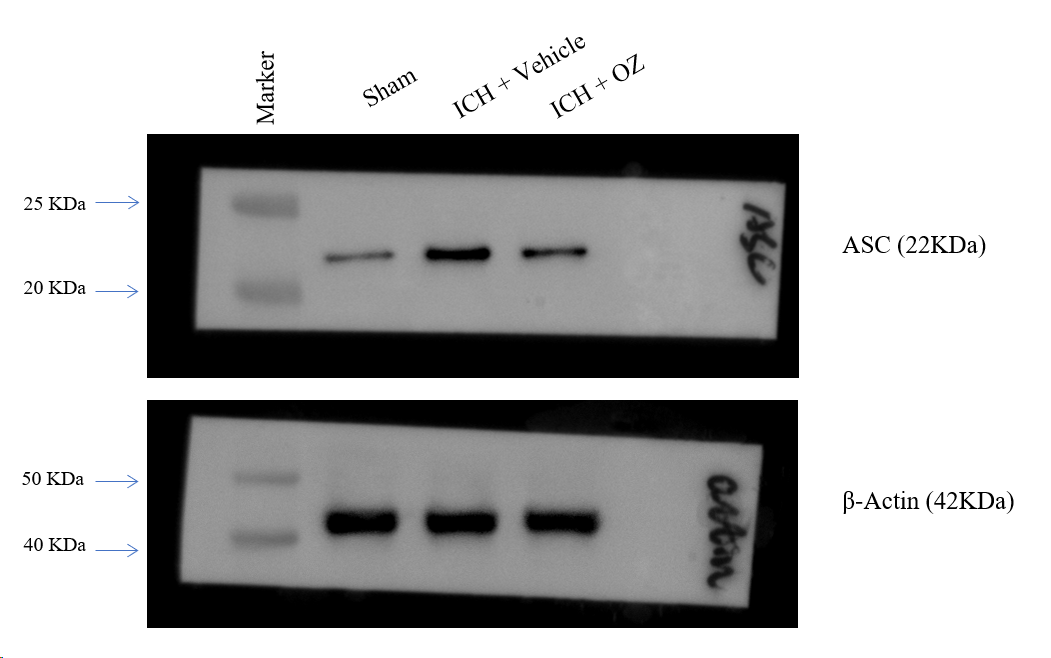
**

**Full unedited gel/blot for Figure 4C in the manuscript.**


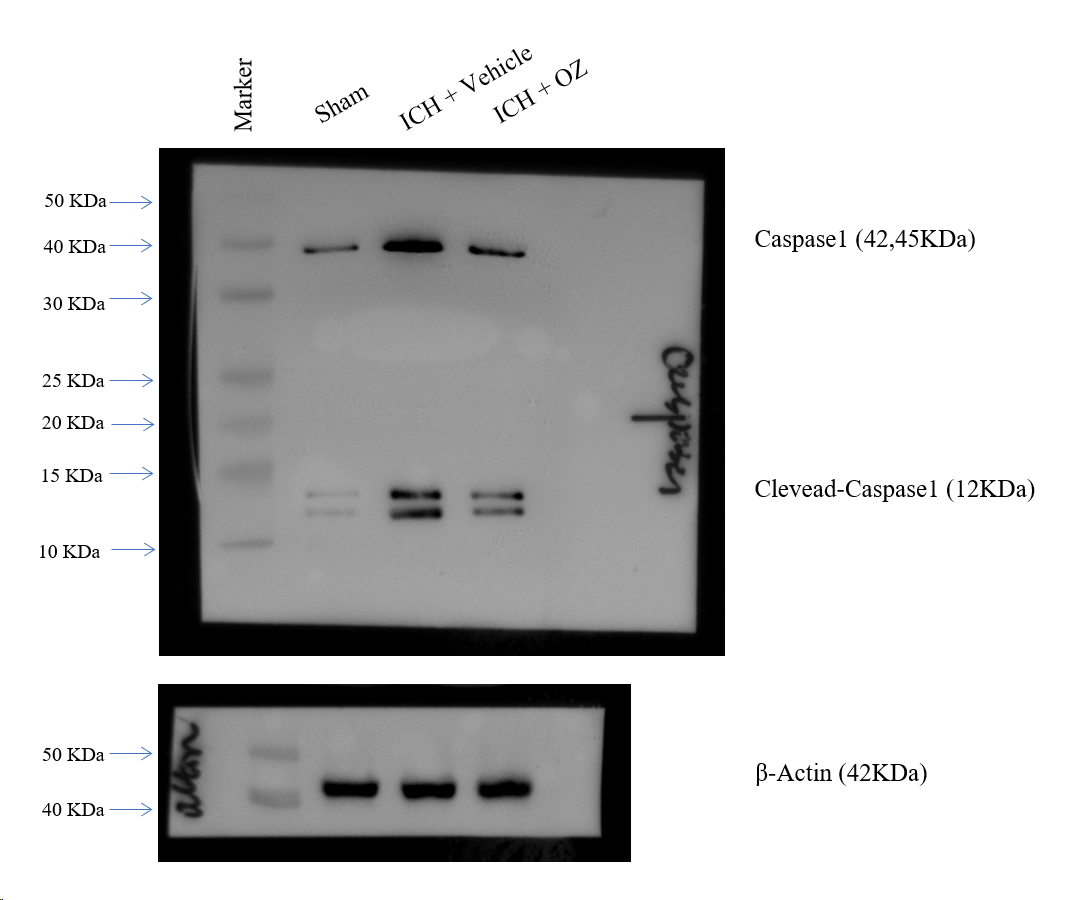


**Full unedited gel/blot for Figure 4D in the manuscript.**

**
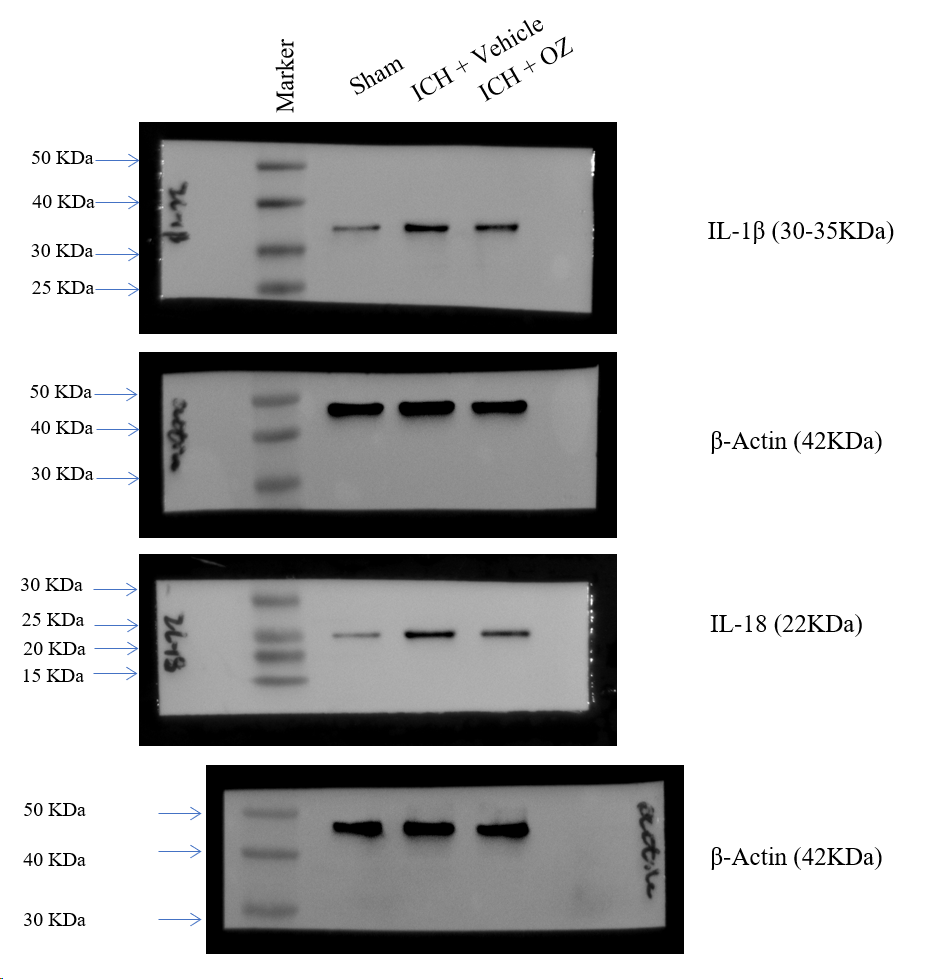
**

**Full unedited gel/blot for Figure5E in the manuscript.**

**
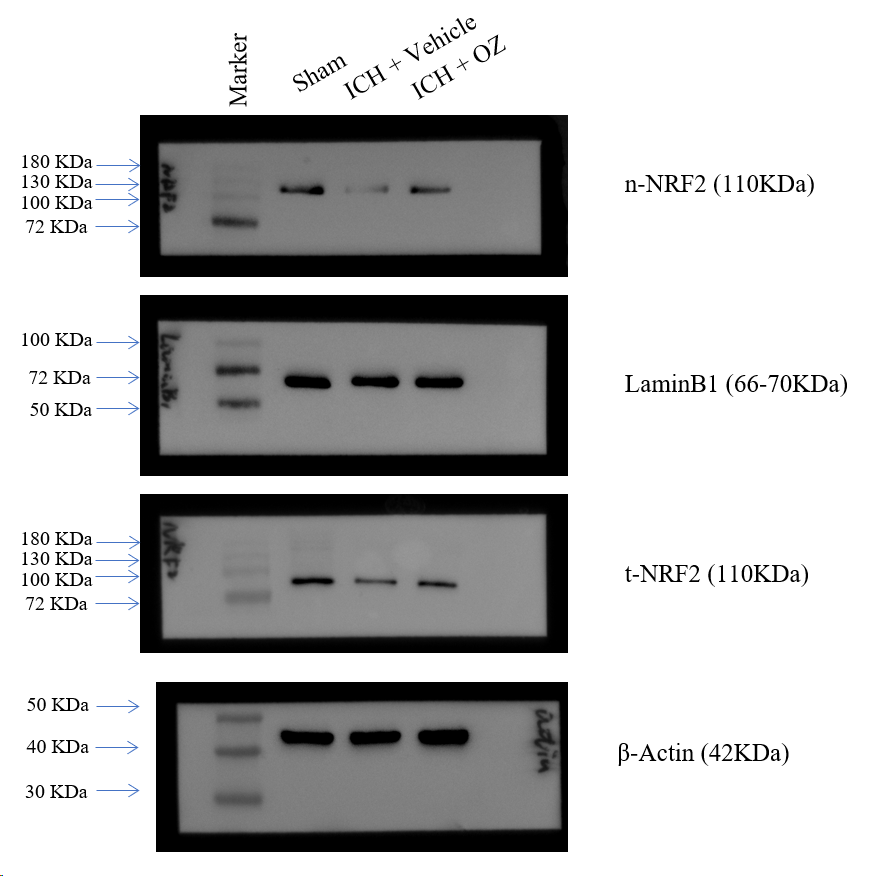
**

**Full unedited gel/blot for Figure5F in the manuscript.**

**
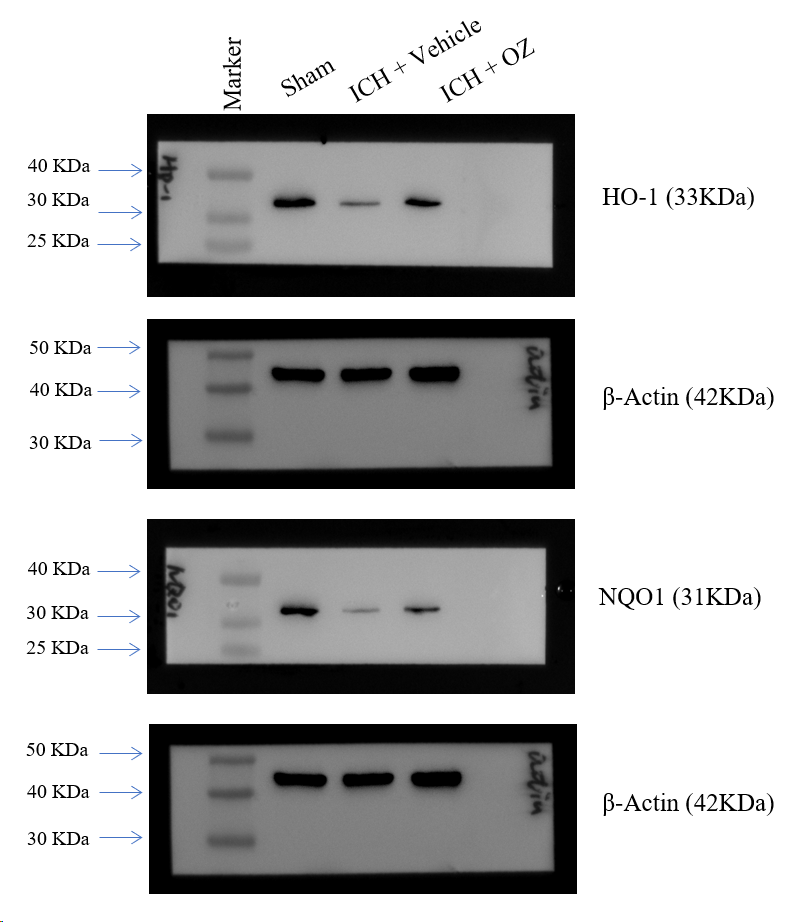
**

**Full unedited gel/blot for Figure5G in the manuscript.**

**
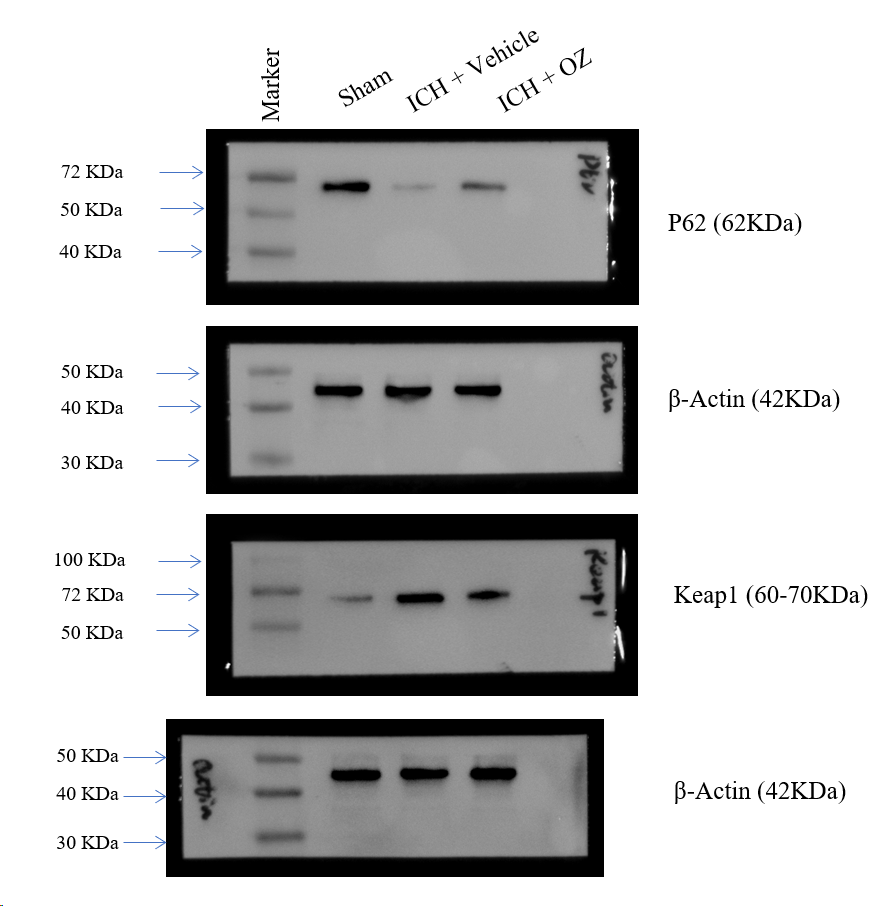
**
