# Supplementary material for: TAK1 inhibition mitigates intracerebral hemorrhage-induced brain injury through reduction of oxidative stress and neuronal pyroptosis via the NRF2 signaling pathway
Source: Front Immunol. 2024 May 2;15:1386780. doi: 10.3389/fimmu.2024.1386780 (PMC11096530; doi:10.3389/fimmu.2024.1386780)
Supplement: Supplementary file 2 [file DataSheet_2.docx]

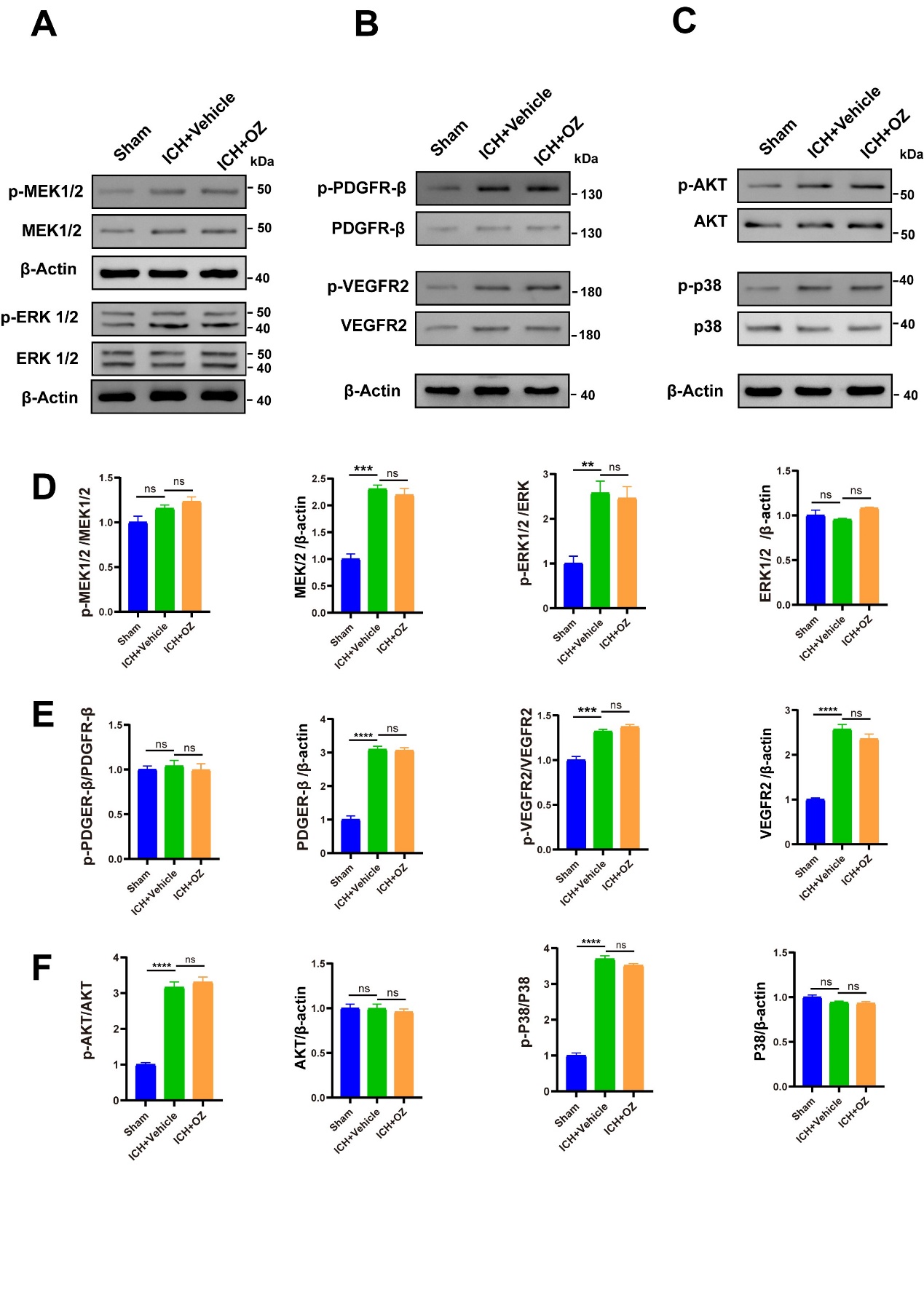


**Fig. S1 The impact of 5Z-7-Oxozeaenol treatment on VEGFR2, PDGFR-β, and MEK1 signaling pathways in the context of ICH.** (A-C) Immunoblots and (D-F) quantitative analysis of p-MEK1/2, MEK1/2, p-ERK, ERK, p-PDGFR-β, PDGFR-β, p-VEGFR2, VEGFR2, p-AKT, AKT, p-P38, P38, and β-actin in Sham, ICH+Vehicle, and ICH+OZ groups. Data are expressed as mean ± SEM, n = 3 in each group. ** *P* < 0.01, ****P* < 0.001 and *****P* < 0.0001 vs Sham group; ns, no significant difference; OZ, 5Z-7-oxozeaenol(1.5μg/μl)
