# Supplementary material for: TAK1 inhibition mitigates intracerebral hemorrhage-induced brain injury through reduction of oxidative stress and neuronal pyroptosis via the NRF2 signaling pathway
Source: Front Immunol. 2024 May 2;15:1386780. doi: 10.3389/fimmu.2024.1386780 (PMC11096530; doi:10.3389/fimmu.2024.1386780)
Supplement: Supplementary file 3 [file DataSheet_3.docx]

**Full unedited gel/blot for Figure S1 in the manuscript.**

The full, unedited gel/blot images for **p-MEK1/2** and **MEK1/2** are provided in Figure S1A of the manuscript.


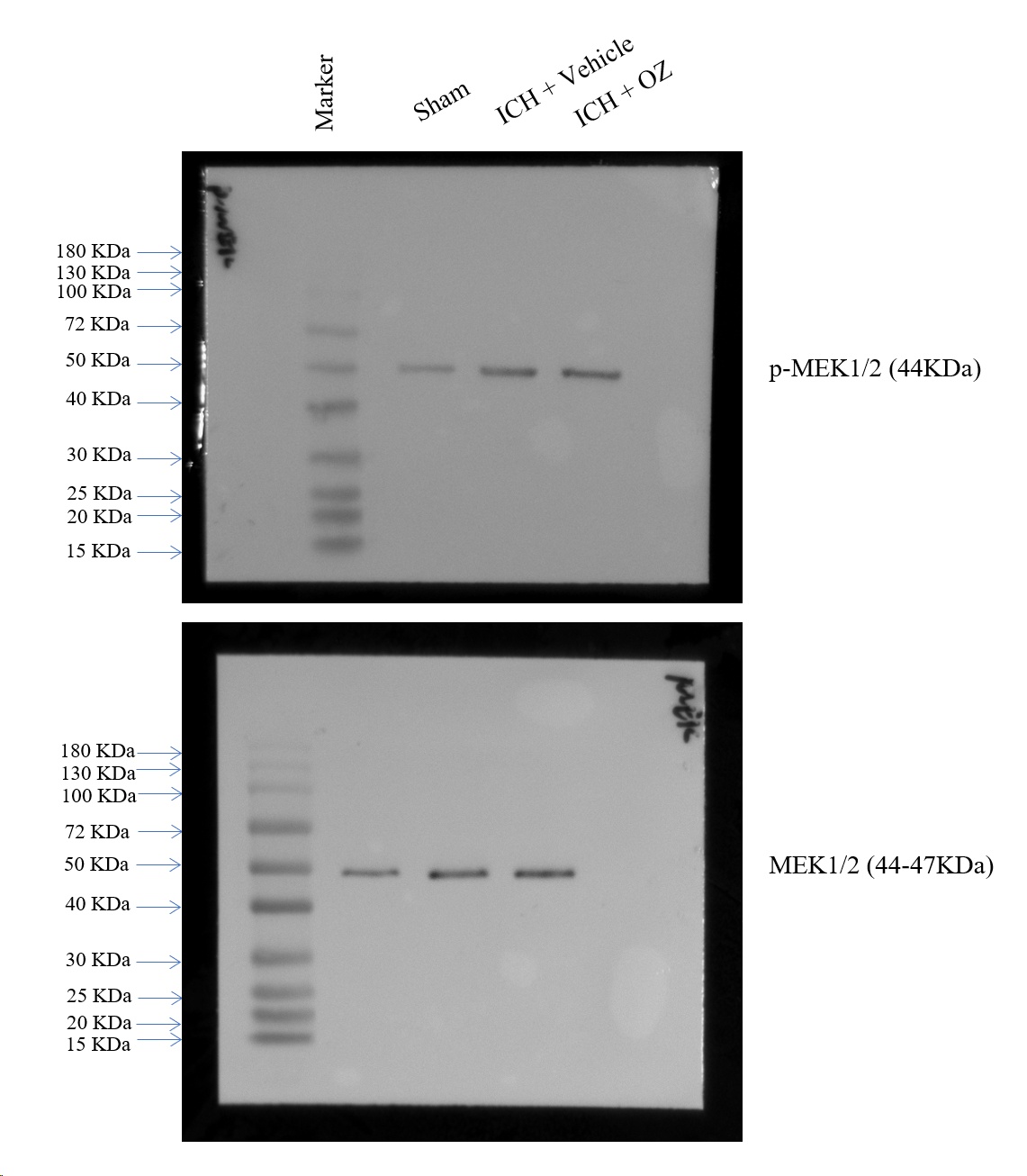


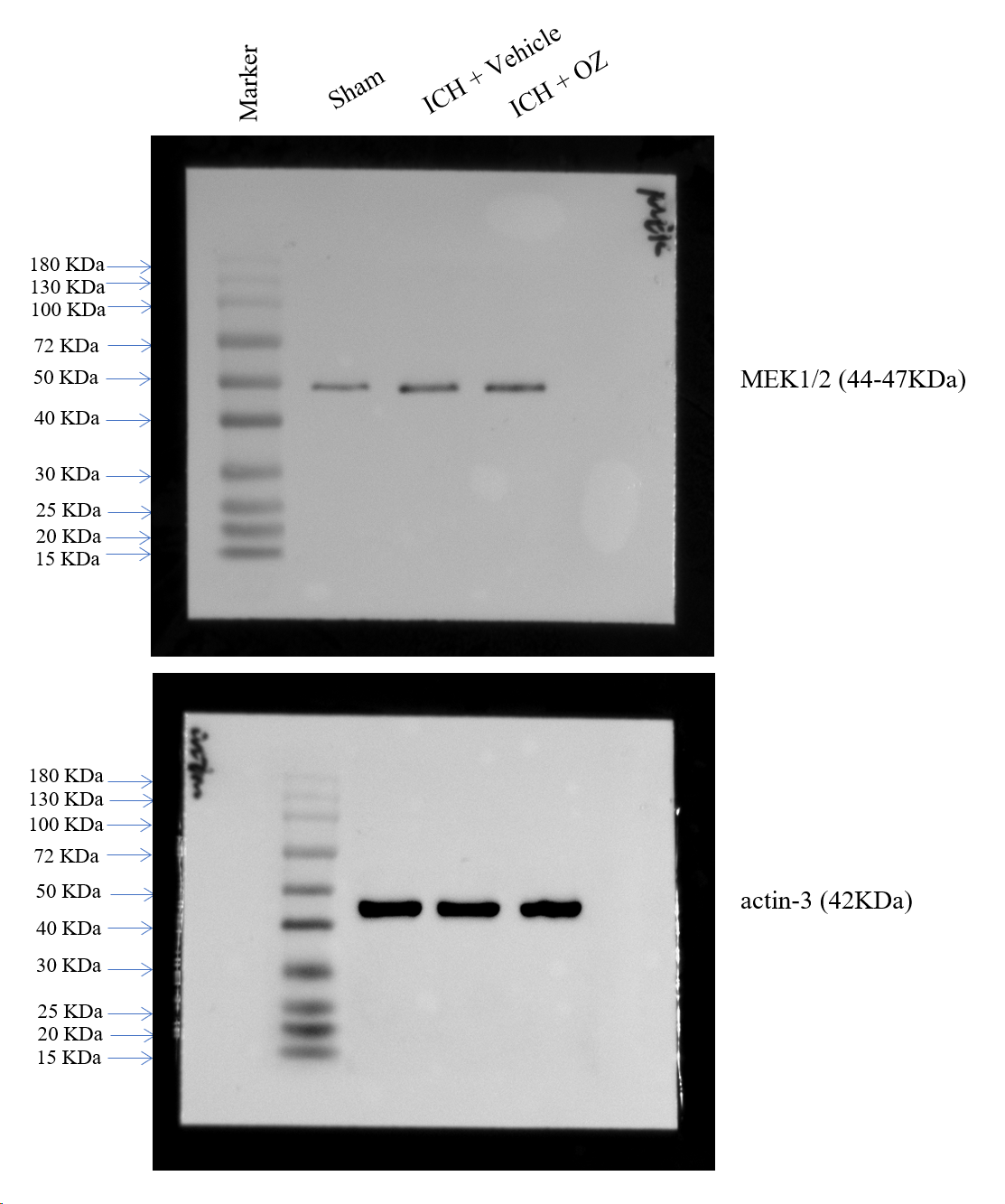


The full, unedited gel/blot images for **p-ERK1/2** and **ERK1/2** are provided in Figure S1A of the manuscript.


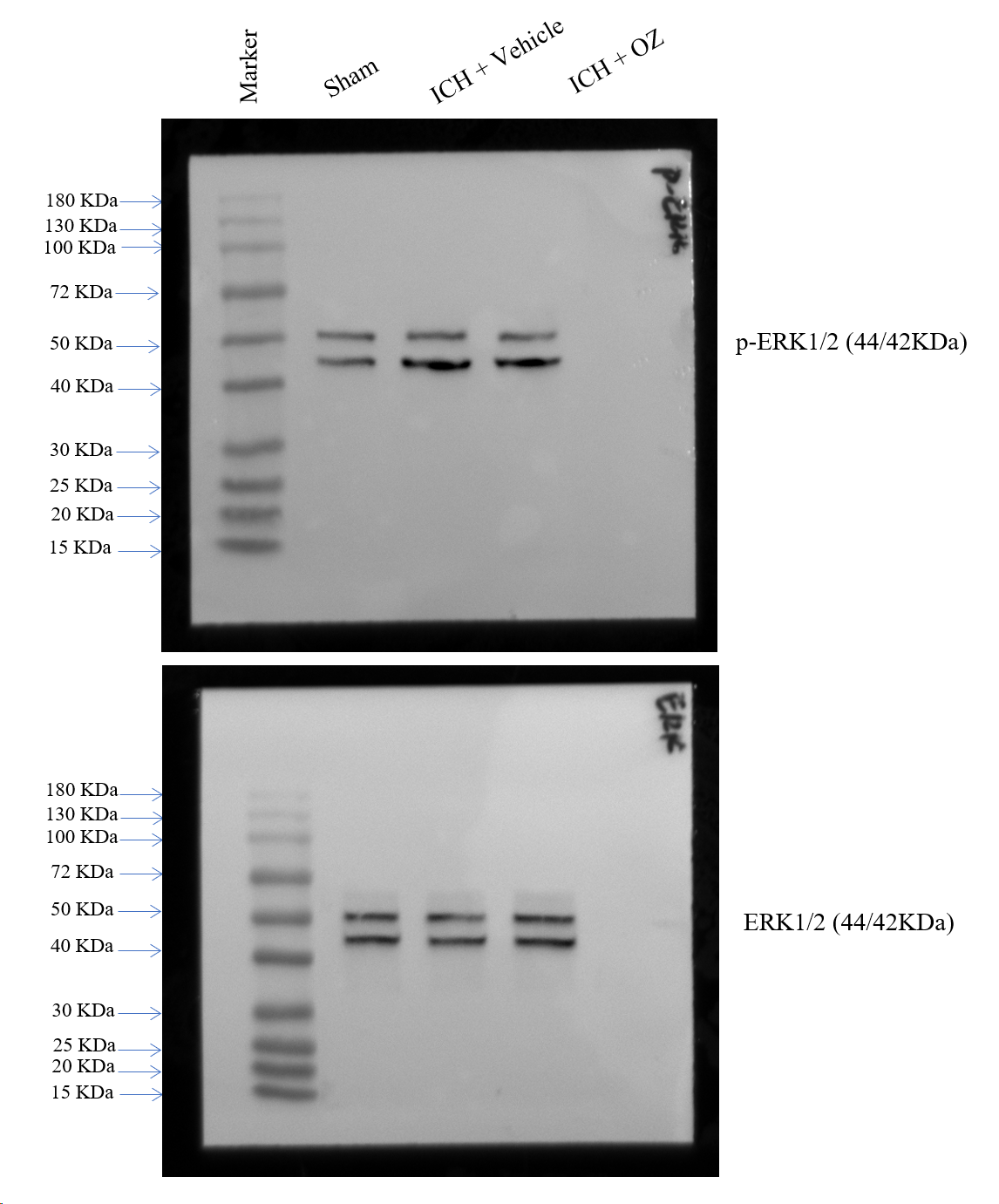


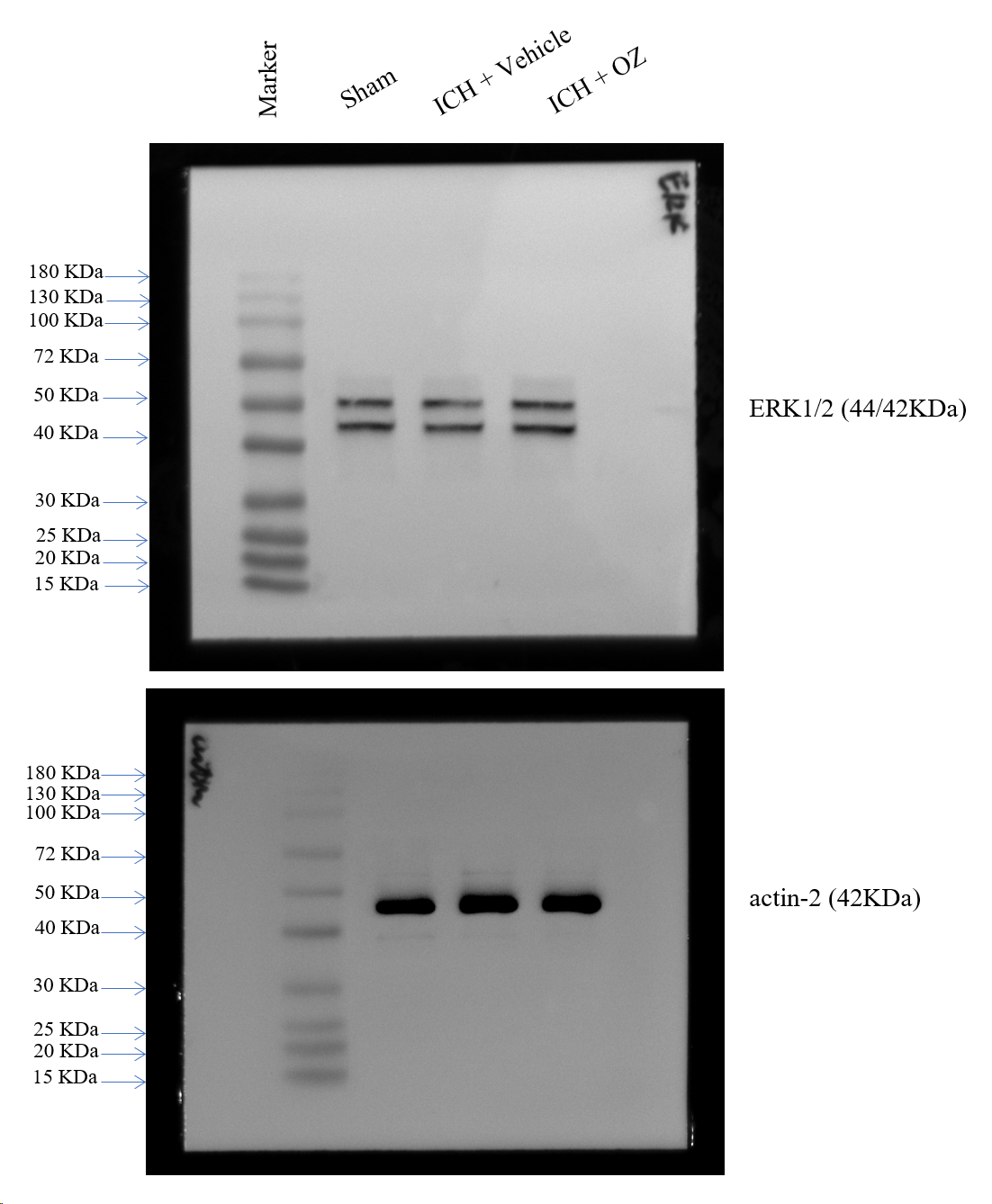


The full, unedited gel/blot images for **p-PDGFR-β** and **PDGFR-β** are provided in Figure S1B of the manuscript.


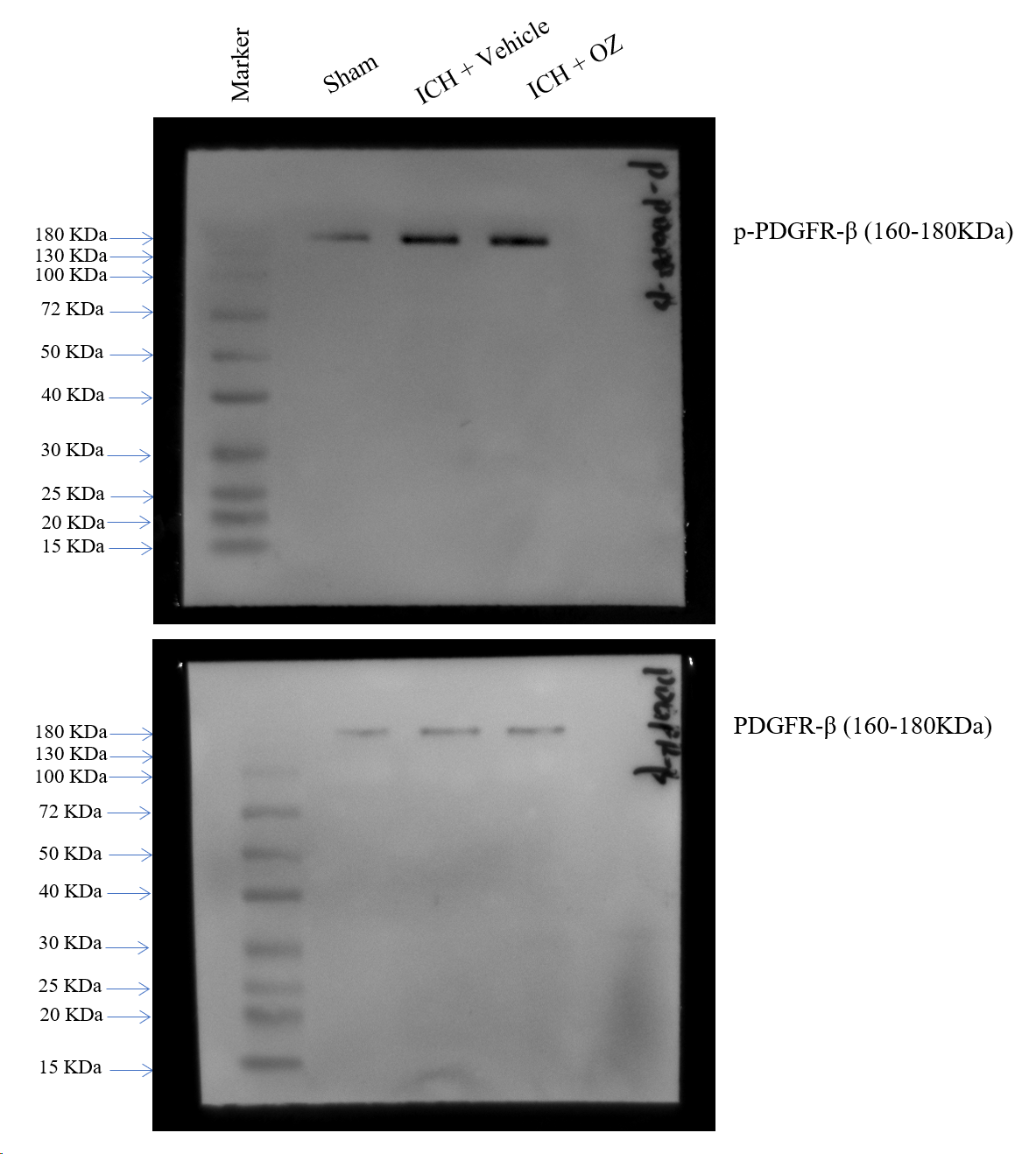


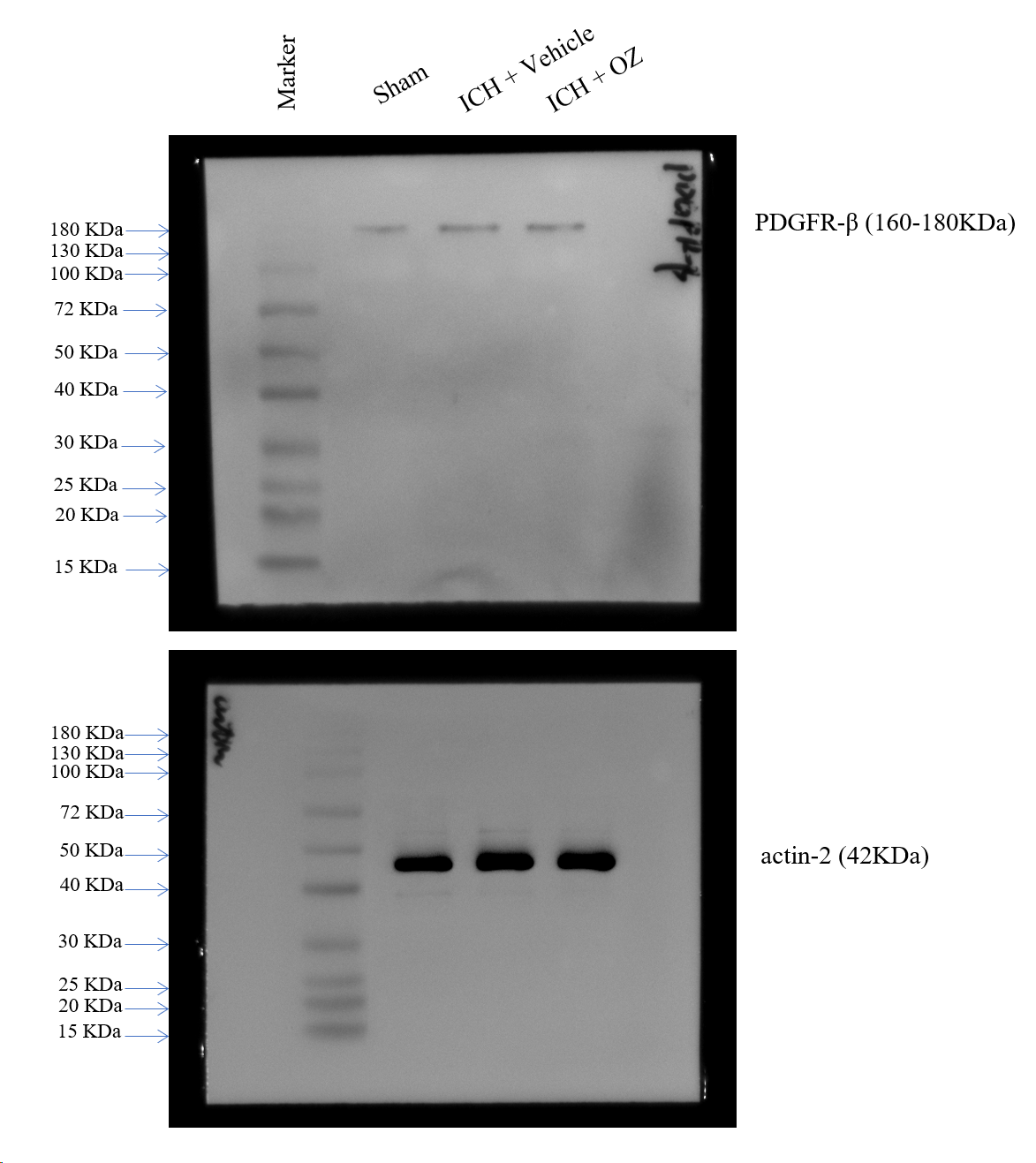


The full, unedited gel/blot images for **p-VEGFR2** and **VEGFR2** are provided in Figure S1B of the manuscript.


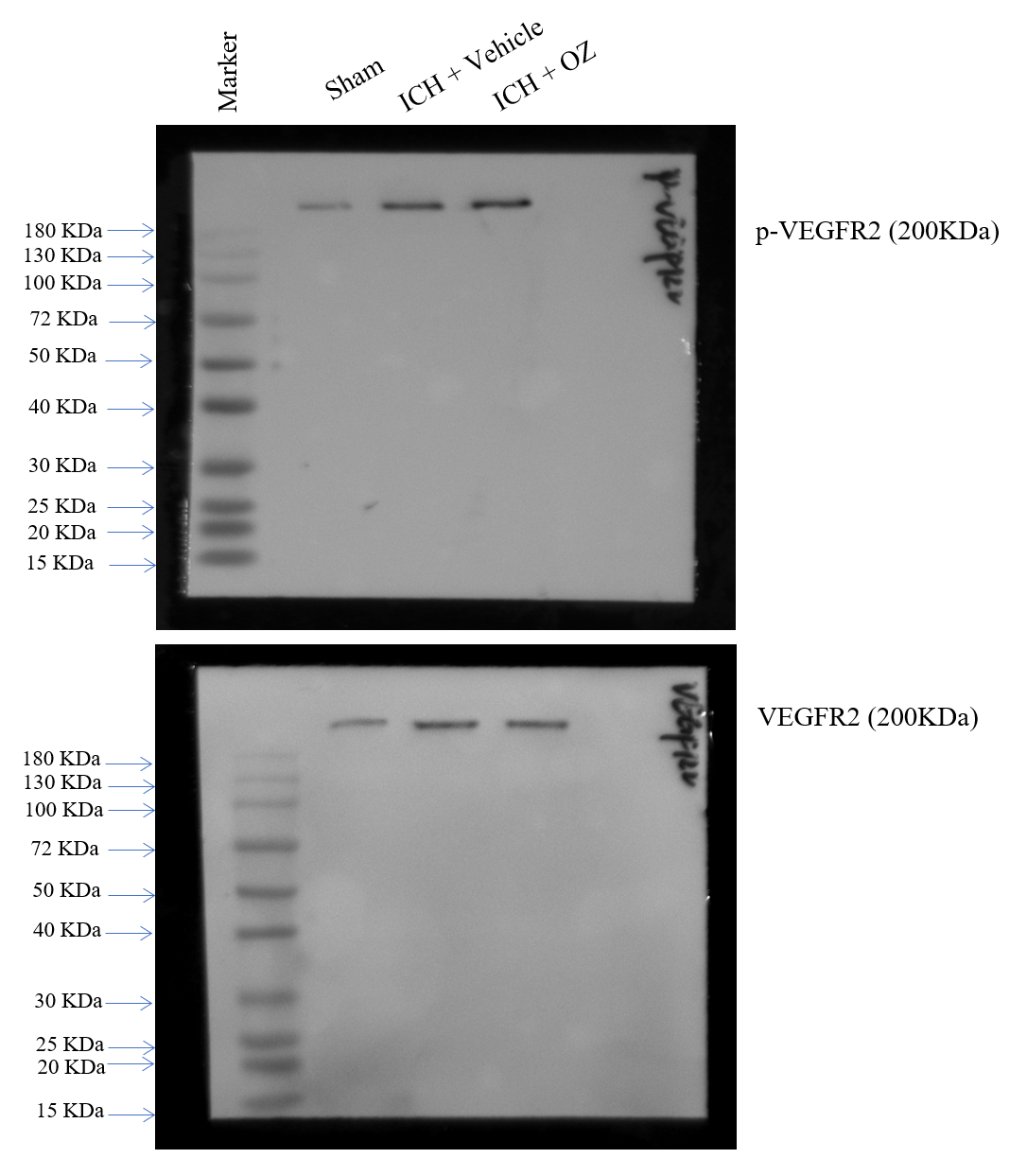


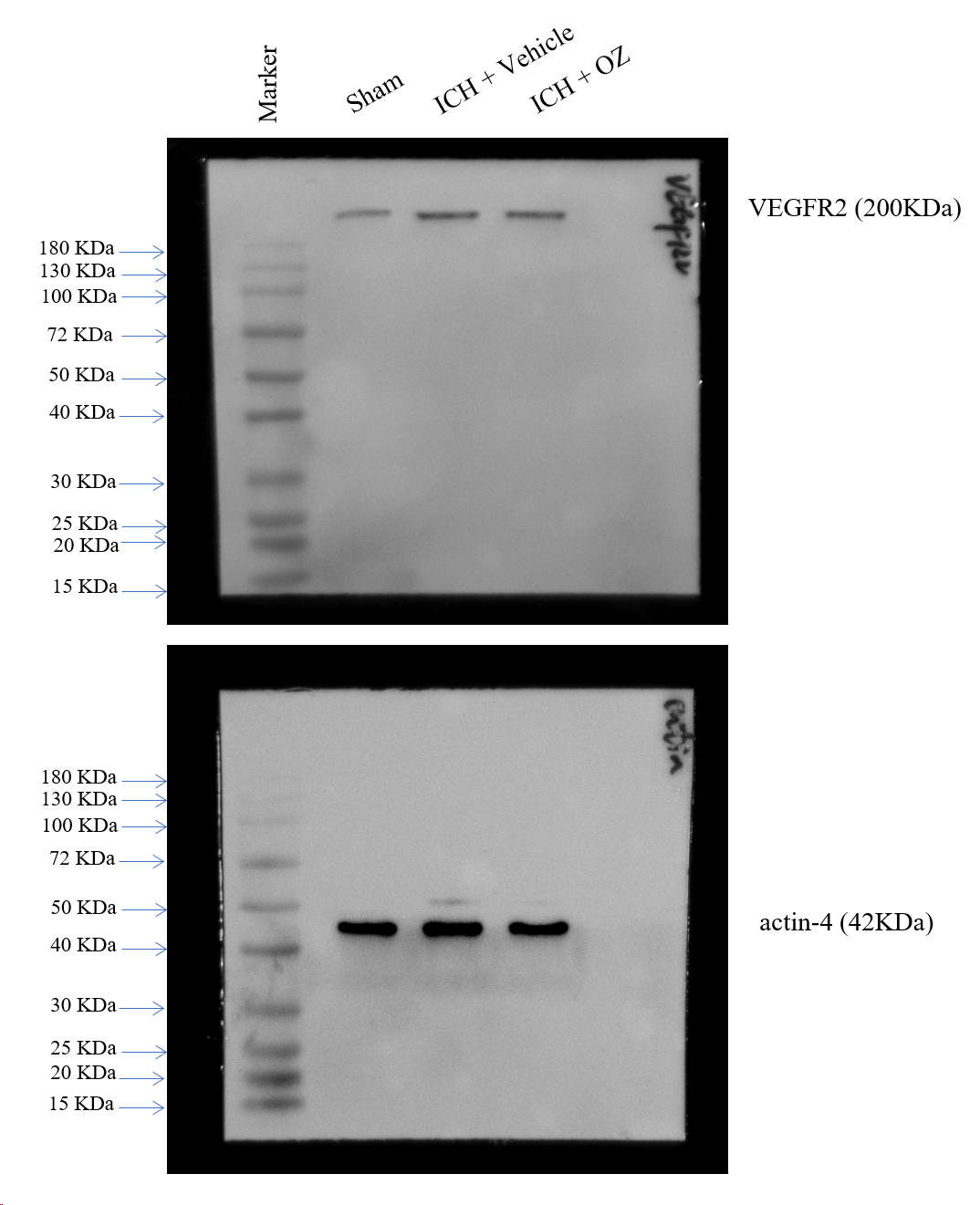


The full, unedited gel/blot images for **p- AKT** and **AKT** are provided in Figure S1C of the manuscript.


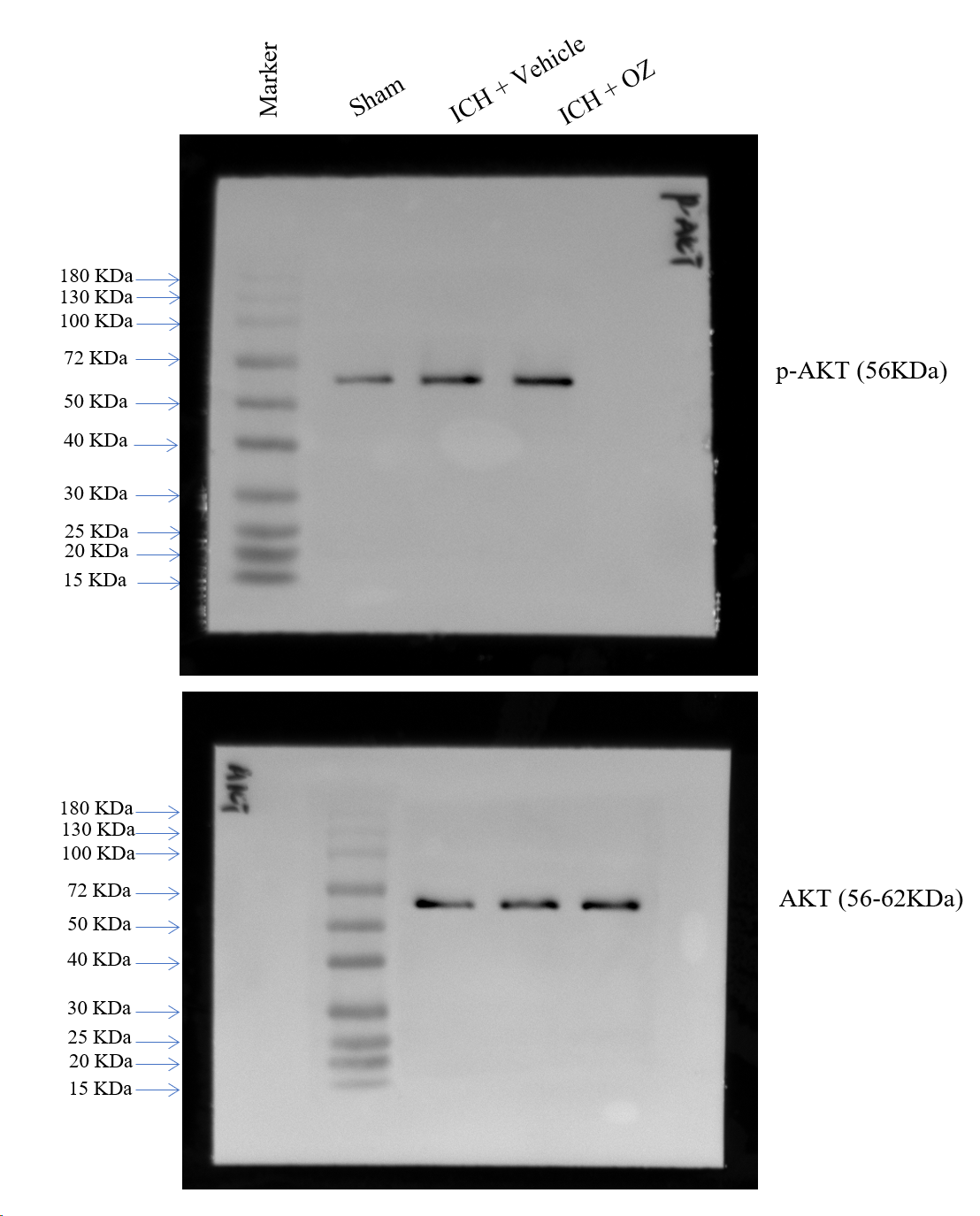


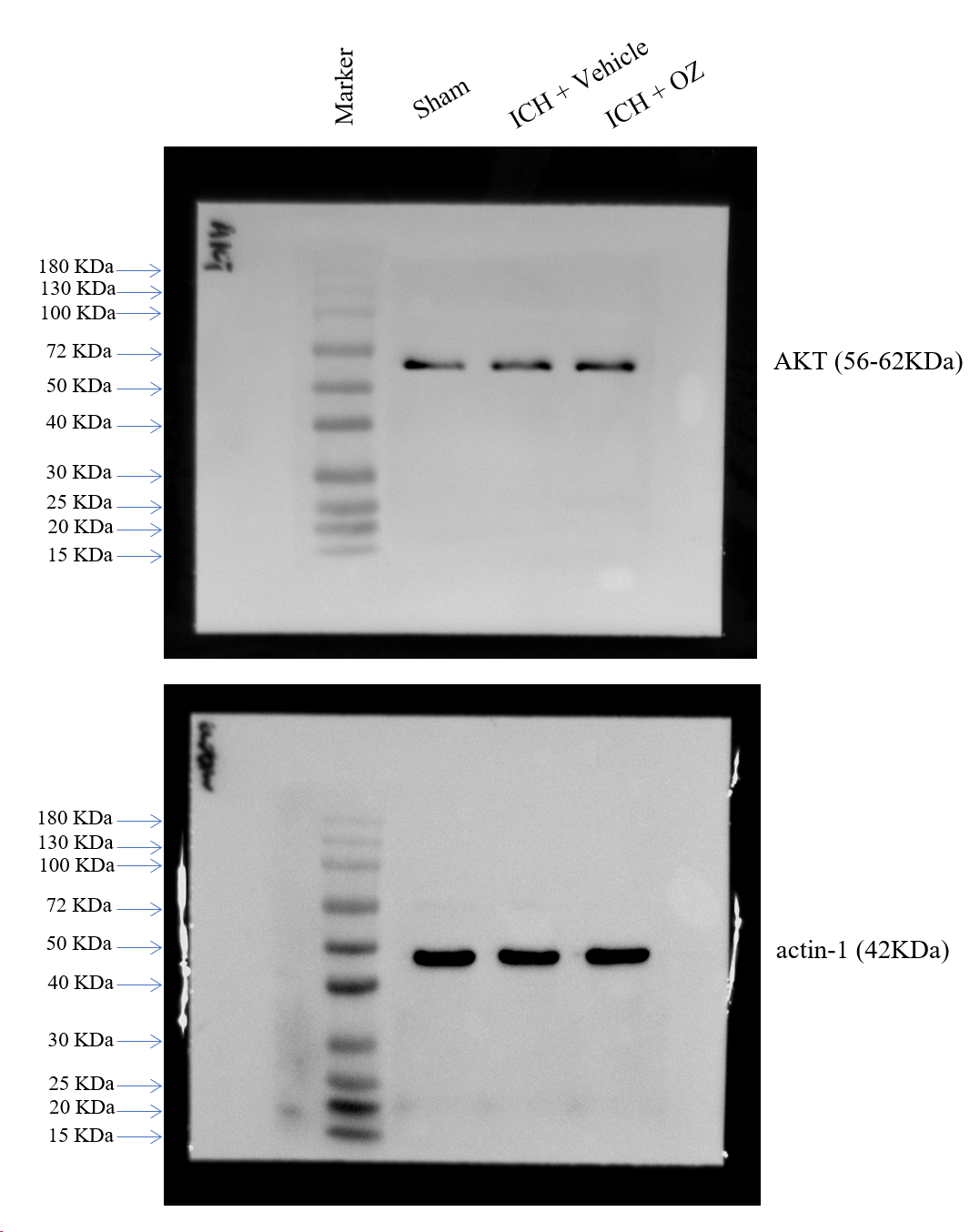


The full, unedited gel/blot images for **p-P38** and **P38** are provided in Figure S1C of the manuscript.


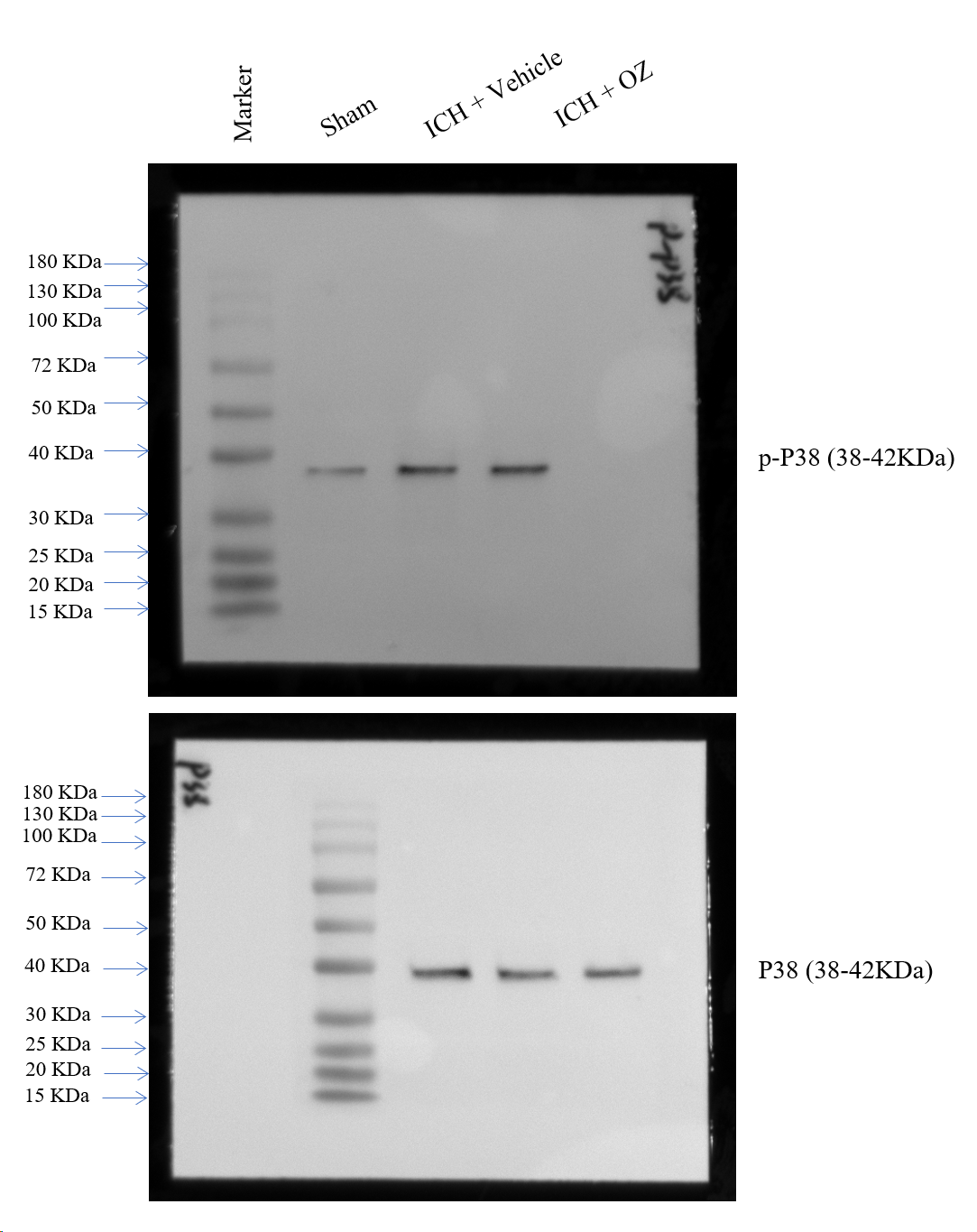


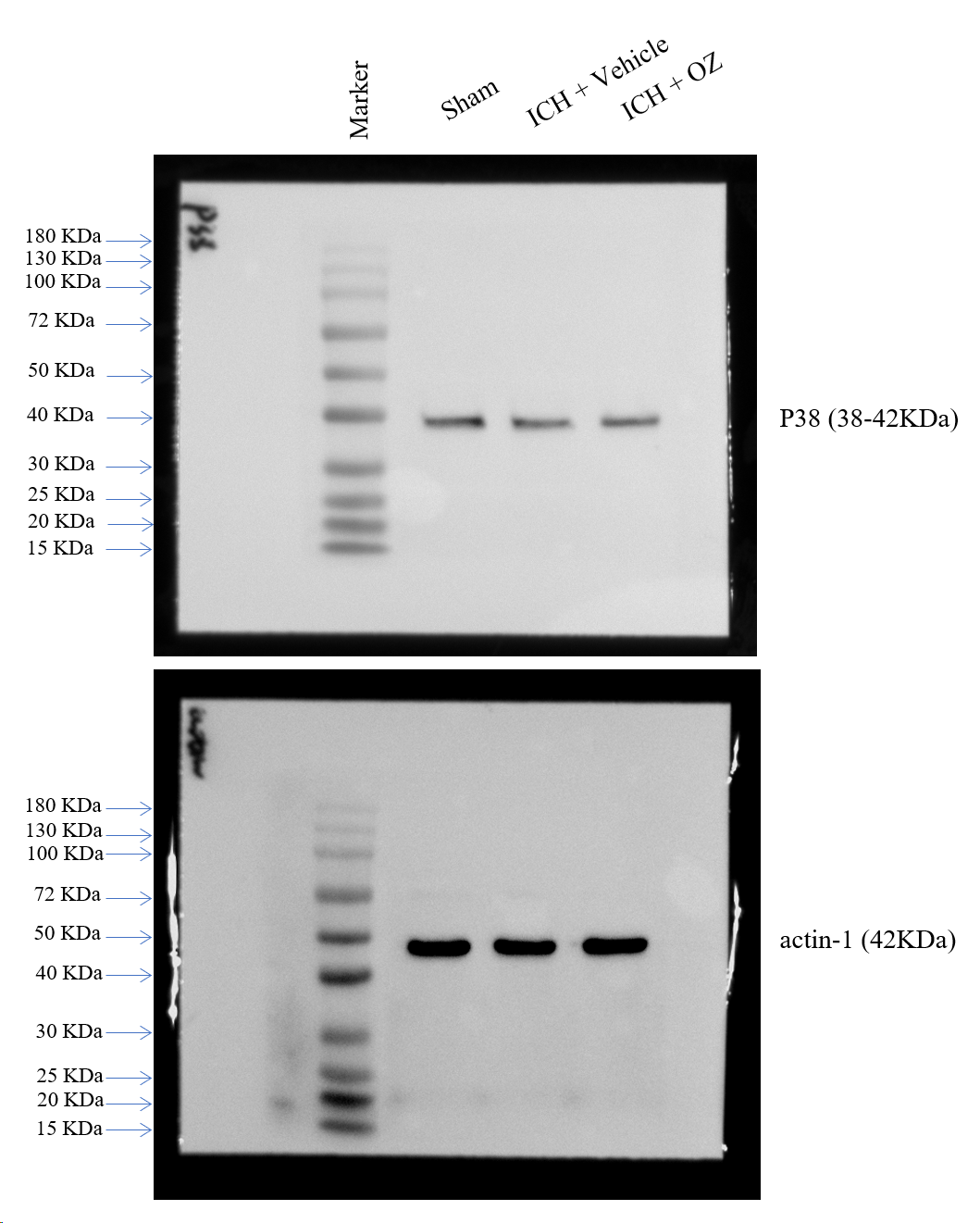


**Key resource table**

| Antibodies | Catalog Number | Host Species | Dilution Ratio | Antibodies Source |
| --- | --- | --- | --- | --- |
| AKT | 60203-2-Ig | Mouse | 1：5000 | Proteintech |
| p-AKT | ab81283 | Rabbit | 1：5000 | Abcam |
| MEK1/2 | 11049-1-AP | Rabbit | 1：5000 | Proteintech |
| p-MEK1/2 | AF8035 | Rabbit | 1：2000 | Affinity |
| P38 | 14064-1-AP | Rabbit | 1：2000 | Proteintech |
| p-P38 | 28796-1-AP | Rabbit | 1：2000 | Proteintech |
| ERK | #4695 | Rabbit | 1：1000 | Cell Signaling Technology |
| p-ERK | #4370 | Rabbit | 1：2000 | Cell Signaling Technology |
| PDGFR-β | 13449-1-AP | Rabbit | 1：1000 | Proteintech |
| p-PDGFR-β | AF3134 | Rabbit | 1：1000 | Affinity |
| VEGFR2 | 26415-1-AP | Rabbit | 1：1000 | Proteintech |
| p-VEGFR2 | AF3279 | Rabbit | 1：1000 | Affinity |
| β-actin | 66009-1-Ig | Mouse | 1：5000 | Proteintech |
