# Supplementary material for: TAK1 inhibition mitigates intracerebral hemorrhage-induced brain injury through reduction of oxidative stress and neuronal pyroptosis via the NRF2 signaling pathway
Source: Front Immunol. 2024 May 2;15:1386780. doi: 10.3389/fimmu.2024.1386780 (PMC11096530; doi:10.3389/fimmu.2024.1386780)
Supplement: Supplementary file 4 [file Table_1.docx]

Supplementary Table 1

Modified Neurological Severity Score

|  | Points |
| --- | --- |
| Motor tests | 6 |
| Raising rat by the tail | 3 |
| Flexion of forelimb | 1 |
| Flexion of hindlimb | 1 |
| Head moved > 10° to vertical axis within 30 s | 1 |
| Placing rat on the floor (normal = 0; maximum = 3) | 3 |
| Normal walk | 0 |
| Inability to walk straight | 1 |
| Circling toward the paretic side | 2 |
| Fall down to the paretic side | 3 |
| Sensory tests | 2 |
| Placing test (visual and tactile test) | 1 |
| Proprioceptive test (deep sensation, pushing the paw against the table edge to stimulate limb muscles) | 1 |
| Beam balance tests (normal = 0; maximum = 6) | 6 |
| Balances with steady posture | 0 |
| Grasps side of beam | 1 |
| Hugs the beam and one limb falls down from the beam | 2 |
| Hugs the beam and two limbs fall down from the beam, or spins on beam  (> 60 s) | 3 |
| Attempts to balance on the beam but falls off (> 40 s) | 4 |
| Attempts to balance on the beam but falls off (> 20 s) | 5 |
| Falls off: No attempt to balance or hang on to the beam (< 20 s) | 6 |
| Absent reflexes and abnormal movements | 4 |
| Pinna reflex (head shake when touching the auditory meatus) | 1 |
| Corneal reflex (eye blink when lightly touching the cornea with cotton) | 1 |
| Startle reflex (motor response to a brief noise from snapping a clipboard  paper) | 1 |
| Seizures, myoclonus, myodystony | 1 |
| Maximum points | 18 |

One point is awarded for the inability to perform the tasks or for the lack of a tested reflex; 13 to 18 indicates severe injury; 7 to 12, moderate injury; 1 to 6, mild injury.
